# Web-Based Tool Designed to Encourage Supplemental Nutrition Assistance Program Use in Urban College Students: Usability Testing Study

**DOI:** 10.2196/50557

**Published:** 2024-06-13

**Authors:** Catherine Yan Hei Li, Charles Platkin, Jonathan Chin, Asia Khan, Jaleel Bennett, Anna Speck, Annette Nielsen, May May Leung

**Affiliations:** 1 Nutrition Program School of Urban Public Health, Hunter College The City University of New York New York, NY United States; 2 Hunter College New York City Food Policy Center Hunter College New York, NY United States; 3 Center For Food As Medicine New York, NY United States; 4 Share Meals Brooklyn, NY United States; 5 Department of Physics and Computer Science Medgar Evers College The City University of New York Brooklyn, NY United States; 6 Public Health Program School of Urban Public Health, Hunter College The City University of New York New York, NY United States; 7 Friedman School of Nutrition Science and Policy Tufts University Boston, MA United States

**Keywords:** SNAP, SNAP eligibility screening, food insecurity, college students, web-based tool, think-aloud, system usability, user experience, student, college, chronic health, stress, anxiety, barrier, technology, tool, Supplemental Nutrition Assistance Program, usability

## Abstract

**Background:**

Food insecurity continues to be a risk for college students in the United States. It is associated with numerous problems, such as chronic health conditions, increased stress and anxiety, and a lower grade point average. After COVID-19, the Supplemental Nutrition Assistance Program (SNAP) benefits were extended to college-aged students; however, there were some barriers to participation, which persisted such as lack of perceived food insecurity risk, lack of knowledge regarding the SNAP application process, the complexity of determining eligibility, and stigma associated with needing social assistance. A technology-enhanced tool was developed to address these barriers to SNAP enrollment and encourage at-risk college students to apply for SNAP.

**Objective:**

The purpose of this study was to test the usability and acceptability of a web-based SNAP screening tool designed for college-aged students.

**Methods:**

College students aged 18-25 years were recruited to participate in 2 rounds of usability testing during fall 2022. Participants tested the prototype of a web-based SNAP screener tool using a standardized think-aloud method. The usability and acceptability of the tool were assessed using a semistructured interview and a 10-item validated System Usability Scale questionnaire. Audio recordings and field notes were systematically reviewed by extracting and sorting feedback as positive or negative comments. System Usability Scale questionnaire data were analyzed using the Wilcoxon signed rank test and sign test.

**Results:**

A total of 12 students (mean age 21.8, SD 2.8 years; n=6, 50% undergraduate; n=11, 92% female; n=7, 58% Hispanic or Black or African American; n=9, 78% low or very low food security) participated in both rounds of user testing. Round 1 testing highlighted overall positive experiences with the tool, with most participants (10/12) stating that the website fulfills its primary objective as a support tool to encourage college students to apply for SNAP. However, issues related to user interface design, navigation, and wording of some questions in the screening tool were noted. Key changes after round 1 reflected these concerns, including improved design of response buttons and tool logo and improved clarity of screening questions. The overall system usability showed slight, but not statistically significant, improvement between round 1 and round 2 (91.25 vs 92.50; *P*=.10, respectively).

**Conclusions:**

Overall usability findings suggest that this web-based tool was highly usable and acceptable to urban college students and could be an effective and appealing approach as a support tool to introduce college students to the SNAP application process. The findings from this study will inform further development of the tool, which could eventually be disseminated publicly among various college campuses.

## Introduction

### Background

Food insecurity in the United States has been studied in many contexts; more recently, there has been a focus on how it affects college students due to recognition of its prevalence in this population. Those enrolled in college are more likely to be food insecure than the average US household, with some studies finding rates of food insecurity as high as 30%, in comparison to the national average of 10.5% [1]. Additionally, because the cost of attending college has increased dramatically and the minimum wage has not risen in concordance with the increased cost of living, working to pay for college and to live is now not feasible for many students [2].

Food insecurity has been associated with poor academic performance, in addition to poorer psychological and physical health [1,3]. There are many coinciding problems that typically accompany food insecurity as well, such as housing insecurity, less access to health care, and other socioeconomic difficulties [3-5]. The use of food assistance programs by college students is less than the nonstudent population due to multiple factors, including lack of self-perceived risk of food insecurity, lack of accommodations and convenience of certain programs, and systemic regulations that impede some college students from accessing federal nutrition assistance programs [2,4,5]. However, there has been more discourse in the political and public health spheres surrounding food insecurity, which may increase students’ access to federal assistance. For example, a law was recently passed in New Jersey that has increased the minimum monthly Supplemental Nutrition Assistance Program (SNAP) benefit from US $50 to US $95, which has addressed the gap that would have been left behind when the pandemic-era benefits were discontinued [6].

The COVID-19 pandemic has affected college students in different ways. Shifting to remote classes and closing campuses resulted in many students changing their residence [1]. However, the more impactful change was the closure of businesses or the reduction of working hours. The COVID-19 pandemic caused 19.7% of college students to experience a reduction in employment after the start of the pandemic, and 18-to-24–year olds had the highest percentage of unemployment among working-age people after the start of COVID-19 [4,7]. These effects were deleterious for those who were already at risk for food insecurity or experiencing food insecurity; yet, only about 4% of eligible college students use nutrition assistance programs [7]. Mitigating factors for students who were at risk of being food insecure or were food insecure before the pandemic include increased unemployment benefits, federal aid available to students through the CARES Act, and moving back in with parents or family [1]. Students who did not relocate for college and were responsible for providing financial support for their family before and during the COVID-19 pandemic were more likely to experience worsened food security [8].

### SNAP Enrollment Among College Students

Current enrollment in SNAP among eligible college students is estimated to be around 30%, providing assistance to 2.26 million students out of 7.3 million who are eligible and in need of food assistance [9,10]. This percentage of eligible students using SNAP has decreased dramatically since the 1990s [2]. Barriers that may explain this reduction in enrollment include a lack of knowledge about requirements and the application process, unnecessarily intricate bureaucracy around the initial application and reapplication of benefits, and strict rules that specifically target college-aged students [2]. The perceived insufficient need is another barrier to applying for students; believing they are ineligible or that they are able to get enough food through other means can prevent them from trying to apply at all. There is also significant stigma surrounding food assistance programs, not limited to SNAP [11]. Many eligible people who are not enrolled in social assistance programs discuss the shame that surrounds experiencing poverty and needing federal or state-funded assistance, in addition to the sense of pride and need to be self-reliant being damaged by enrolling in government-funded programs [11].

Facilitators to college student enrollment in SNAP largely revolve around increased support available on and near campus for learning about the application process and providing tools to support the application process. Essentially, providing support and information about the program and its application process and increasing awareness of eligibility requirements to combat perceived insufficient need, alongside destigmatizing the use of government assistance programs, should help increase SNAP enrollment among eligible college students [12]. Other screening tools for New York residents have been developed both by independent organizations—such as the Community Service Society of New York’s screener and Hunger Solutions NY [13] and by public offices, such as NYC’s ACCESS tool [13-15]. However, these tools do not target college students specifically and often do not offer information about student-specific eligibility requirements, highlighting a gap in available resources for a population that experiences food insecurity at higher-than-average rates.

### Study Objective

Guided by formative research and user-centered design approaches, an educational web-based tool prototype that provides information about the SNAP application process and a brief SNAP eligibility screener was developed. The objective of this study was to evaluate the perceived usability and acceptability of this web-based tool among urban college students.

## Methods

### Study Overview

This mixed methods study was conducted from September to December 2022 at a 4-year public college in New York City. Two sessions of usability testing were conducted using a standardized think-aloud method. Both qualitative and quantitative data were collected.

### Ethical Considerations

The protocols of this study were reviewed by the City University of New York Human Research Protection Program. The study was determined to not be defined as research, per institutional guidelines. Therefore, formal institutional review board approval of its procedures was not needed (protocol 2022-0508). However, oral consent was still obtained from the participants prior to the initiation of any study procedures. Participants also had the ability to opt out of the study at any point in time. Compensation was provided for study participation. Specifically, participants received up to US $30 in Amazon gift cards (US $10 and US $20 for round 1 and round 2, respectively) upon completion of the usability testing sessions. All analyses were conducted with deidentified data.

### Participants

English-speaking college students aged 18 to 25 years were recruited to participate in 2 rounds of usability testing to provide feedback and identify problems to help inform the development of the web-based tool. Participants were recruited via flyers placed in communal spaces of campus buildings and emails that were sent to students through departmental email lists, which contained a digital copy of the flyer and sign-up link. The flyers contained general study information, including study purpose, compensation, location, eligibility, and contact information, and a QR code linking to the screening questionnaire. Prospective participants were able to access a self-administered screening questionnaire to screen for eligibility. Inclusion criteria also included students who were at risk for food insecurity or who answered yes to not having enough money for food within the past 30 days. A total of 50 potential student participants completed the screening survey, and 33 (66%) were found to be eligible. However, only 12 agreed to participate in usability testing sessions, which is an expected sample size for such types of studies [16-21]. The remaining 21 students became uncontactable, declined to participate in the study, or failed to schedule a usability testing session.

### Website Design and Features

The development of this screening tool prototype was guided by constructs from the social cognitive theory and the theory of planned behavior. The social cognitive theory characterizes learning as a process that is affected by dynamic interactions between the individual, environment, and behavior [22]. The theory of planned behavior posits that intention to perform a behavior is influenced by attitude toward the behavior, normative and subjective beliefs, and perceived behavioral control [23]. The contents of the web-based tool were developed to address the constructs of behavioral capability, outcome expectations, attitudes, subjective norms, perceived control, and self-efficacy. The focus of the tool was to provide information to clarify the intricacies of the SNAP application process and empower college students to submit applications so that more eligible students would receive and use SNAP benefits.

The design of this web-based tool was grounded in user-centered design approaches to create a convenient, appealing, and straightforward website to promote potential engagement [24,25]. Development began with defining the target audience and constructing prototypical profiles of potential college student users, which generated ideas on what expectations users would have when using an informative SNAP website. The research team also analyzed other similar websites, including government-affiliated SNAP websites and other SNAP eligibility screening tools to identify gaps in service to urban college students. This formative research found that while existing tools provided varying levels of detail about SNAP for the general adult population, there was a lack of easily accessible information specific to the college student population. Furthermore, during usability testing, participants were encouraged to provide solutions to perceived usability issues, facilitating user input to inform the continual development of the tool.

**Figure 1:**
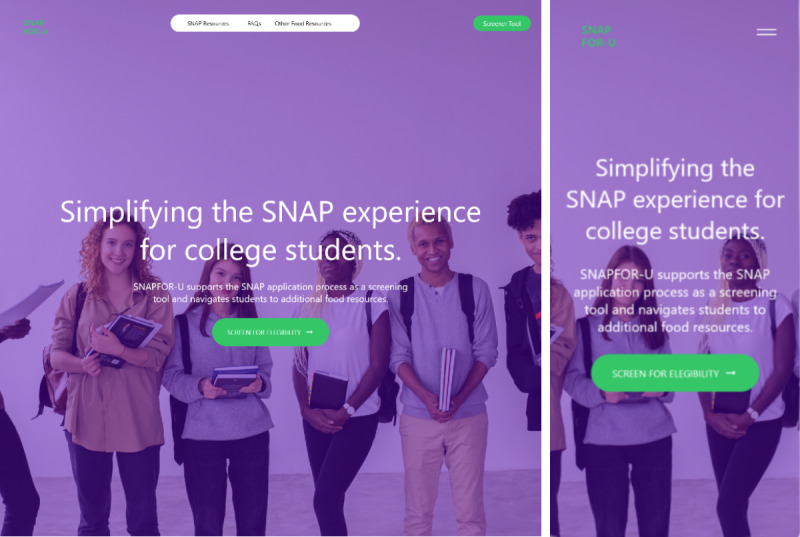
Round 1 home screen of the SNAP For-U webpage on desktop (left) and mobile devices (right).

SNAP For-U was developed as an educational web-based tool, offering detailed information about the SNAP application process and a quick SNAP eligibility screener (Multimedia Appendix 1). The website contained 3 text-based informative content pages, in addition to the more visual-focused home page (Figure 1). The home page included colorful imagery, positive messaging, facts, and testimonials from college students. These were included to address the stigma surrounding SNAP use by showing perspectives that oppose normative beliefs on self-reliance and to show how SNAP can have a positive impact on a recipient’s livelihood. Other pages contained more detailed text with referrals to resources to assist with applying for SNAP and information to questions a college student may have when applying, which aimed to provide instruction and guidance. An additional page was added to provide other food assistance resources for users who choose not to apply or are found to be ineligible for SNAP. These pages objectively lay out information about SNAP to avoid associations of shame and other negative perceptions. The SNAP screening tool consisted of questions relating to eligibility criteria assessed on the SNAP application, including questions specifically pertaining to the additional eligibility restrictions students often face. The results page presents an estimation of eligibility and monthly benefit amount. This screening component aims to enhance self-efficacy by serving as a preview to the SNAP application and improve outcome expectations as receiving a favorable outcome on the results page could encourage students to engage in the perceived riskier and more time-consuming process of submitting a SNAP application. The highlighted eligibility factors and application guidance also convey the aspects of the SNAP application that are within or outside of the applicant’s control to increase agency and manage expectations and perceived behavioral control.

### Data Collection

#### Usability Sessions

Testing sessions for round 1 were conducted from September to October 2022 and round 2 sessions were conducted from November to December 2022 in a private room in a college campus building with 3 trained researchers (CYHL, AK, and AS), which included a moderator and a note taker. The option of participating in usability sessions remotely via Zoom (Zoom Technologies) was also offered to a portion of students to provide flexibility in scheduling due to some prospective participants expressing difficulty in attending in-person sessions. Although participants were not required to use a camera for remote sessions, participants were asked to share their screens for the entirety of testing to allow researchers to better document their natural workflow of the tool.

Baseline sociodemographic data, including household SNAP use, food security status, and technology and internet access, were collected during round 1 sessions. During the usability testing sessions, a combination of qualitative and quantitative methods was implemented. This was essential in following the iterative design cycle [26]. Each session began with a brief think-aloud training, where participants demonstrated their understanding of the think-aloud method. Participants were asked to pay attention to what they were looking at and verbalize their thoughts in detail. Following the think-aloud procedure, the moderator followed up with guided open-ended questions to allow participants to share any feedback and recommendations for improvement of the tool. Participants accessed the web-based tool using the device of their choice (laptop, tablet, or phone) as it allowed participants familiarity with the device. All usability testing sessions were audio-recorded and the note taker documented the participant’s comments, performance, behaviors, and nonverbal body language.

#### Think-Aloud Procedure

Think-aloud protocols are a widely used method for the usability testing of software, interfaces, websites, and mobile apps [27]. The basic principle of the think-aloud process is that potential users are asked to complete a set of actions and constantly verbalize their thoughts while navigating through these actions. A combined concurrent and retrospective methodology remains especially effective in illustrating users’ cognitive processes while navigating databases and the web [28-30]. Concurrent think-aloud is effective for the collection of a user’s candid thought processes as they engage with a tool in real time, but verbalizing perceptions may feel unnatural to some users which could cause users to cognitively process information differently than if the tool were to be used in a real-world setting [29]. Retrospective methods probe for users to reflect upon their experience after product engagement, allowing users to express their perspectives from a holistic view of their overall user experience [29]. In this study, research assistants asked participants to use a prototype of the website. Participants were first introduced to the website through its home page. From the home page, participants accessed the messaging, resource pages, and screening tool freely. Participants were prompted to interact with each of these sections while continuously verbalizing their thoughts and actions on functionality. Once the think-aloud process began, participants navigated the tool with minimal intervention from research personnel [31]. The moderator followed a guide with suggested questions, feedback, and words of affirmation to encourage all reactions and thoughts from participants to be verbalized throughout testing. If participants stopped talking during this procedure, research personnel briefly reminded them to keep talking using only short, indirect phrases, such as “What are you thinking right now?” “How do you feel about this?” Prior to beginning the think-aloud session, each interview began with the research assistant explaining the concept of the think-aloud protocol (eg, “I want you to say what you are doing and thinking as you navigate through the different sections of the website”). A demonstrative example was given by the research assistant by going through the steps of setting an alarm following the think-aloud protocol. After the tutorial, participants were then asked to practice the protocol themselves. They were given two options: (1) to change the wallpaper on their selected device or (2) to take a 5-minute animal personality quiz [32]. After each session, participants demonstrated an understanding of the think-aloud protocol and were then introduced to the website protocol.

During the latter half of the session, participants were asked a series of open-ended questions to allow participants to reflect on their user experience and elaborate on feedback or comments given during the testing. Participants were allowed to interact with the tool during the questions to show which aspects of the tool they were referring to. The questions asked pertained to overall impressions, visual design and format, navigation, content and usability of each feature of the tool, and overall perceived acceptability. Participants were reminded that there were no correct or incorrect answers and that the questions were an opportunity to express how usable, acceptable, and effective the tool was so that it could be improved.

#### Perceived Usability and Acceptability

The usability and acceptability of the web-based tool were assessed through a quantitative questionnaire and a semistructured interview. The validated System Usability Scale (SUS) questionnaire was administered to participants via pen and paper [33]. The SUS questionnaire contains 10 items of alternating positive and negative statements rated on a 5-point Likert scale from 1 (strongly disagree) to 5 (strongly agree). An additional question was added to the questionnaire to ask participants to rate their likelihood of recommending the tool to others on an 11-point Likert scale (0 to 10) to assess perceived acceptability.

### Data Analysis

#### Content Analysis

Deidentified data from the think-aloud procedure and interview questions were systematically analyzed by 3 reviewers (CYHL, AK, and AS) in a streamlined process guided by methodology from summative content analysis approaches [34,35]. The overall process consisted of listening to audio recordings and then extracting relevant feedback, comments, or responses to a matrix sheet to create summaries of user experiences. Audio recordings from usability sessions were not transcribed verbatim to preserve the nonverbal contextual cues (eg, vocal tones and tapping of device screens). A rapid data analysis technique informed by REAM (Rapid Evaluation and Assessments Methods) and RITA (Rapid Identification of Themes From Audio Recordings) was used to summarize participant feedback and to identify relevant key quotations [36,37]. The use of qualitative data analysis software and coding was determined to not be necessary for this streamlined process. Microsoft Excel (version 16.01) was used to organize and analyze the data.

Before conducting the analysis, 3 independent reviewers identified general categories to construct the matrix sheet by discussing field notes briefs. The categories included the overall impressions, visual design and format, navigation, the specific components of the website, personal SNAP experiences, and other feedback. Field notes were initially reviewed to provide an overview of a usability session and inform the direction for further analysis. Audio recordings and field notes were then reviewed simultaneously and summarized to capture all positive, negative, and neutral feedback or suggestions. Reviewers also extracted keywords or phrases and quotations that characterized the user’s experience. Themes emerged as reviewers noted redundancy between rounds and convergence of comments between participants. After the summarization of data, instances of similar feedback being conveyed by multiple participants were aggregated. Throughout the analysis process, reviewers discussed with each other to continually validate the extraction and grouping of data, and resolve discrepancies.

#### Quantitative Data

Quantitative data from the SUS questionnaire were managed in Microsoft Excel (version 16.01) to score individual SUS questionnaire responses and calculate the overall SUS score. Further analyses were conducted in SPSS (version 26; IBM Corp). Descriptive statistics were used to report averages and SDs. Comparisons of usability scores between rounds were done using the Wilcoxon signed rank test and sign test.

## Results

### Participant Characteristics

A total of 12 students participated in usability testing sessions. Round 1 consisted of 8 in-person sessions and 4 remote sessions via Zoom. Participants were asked to maintain the same modality for round 2 sessions, but 1 participant switched from in-person to remote due to a scheduling conflict. Demographic characteristics of the study sample are summarized in Table 1.

**Table 1:** Sociodemographic characteristics of participants (N=12).

Characteristic		Value
Age (years), mean (SD)		21.8 (2.8)
**Sex, n (%)**		
	Male	1 (8)
	Female	11 (92)
**Ethnicity, n (%)**		
	Asian or Pacific Islander	3 (25)
	Black or African American	2 (17)
	Hispanic or Latinx	5 (42)
	White	2 (17)
**Born in the United States, n (%)**		
	Yes	9 (75)
	No	3 (25)
**Level of study, n (%)**		
	Undergraduate	6 (50)
	Graduate	6 (50)
**School, n (%)**		
	School of Arts and Sciences	3 (25)
	School of Social Work	3 (25)
	School of Urban Public Health	5 (42)
	Undeclared	1 (8)
**Household composition, n (%)**		
	One person	2 (17)
	With family members	9 (75)
	With a spouse or partner	1 (8)
**Annual household income (US $), n (%)**		
	Below $30,000	3 (25)
	$30,000-49,999	6 (50)
	$50,000 or more	3 (25)
**Household currently receives SNAP^a^, n (%)**		
	Yes	3 (25)
	No	7 (58)
	I do not know	2 (17)
**Food security, n (%)**		
	High or marginal	3 (25)
	Low	5 (42)
	Very low	4 (33)
**Devices used to access the internet, n (%)**		
	Desktop computer or laptop	10 (83)
	iPad or tablet	4 (33)
	Smartphone	12 (100)

^a^SNAP: Supplemental Nutrition Assistance Program.

The mean age of participants was 21.8 (SD 2.8) years, and almost all of the participants (11/12, 92%) identified as female. Participants were from a number of cultural backgrounds, with the majority (10/12, 83%) being of an ethnicity other than White. Furthermore, in addition to English, the majority of participants (8/12, 67%) spoke other languages. Half of the participants reported annual household incomes within the range of US $30,000 to US $49,999. Of 12 participants, only 3 (25%) participants attested to currently receiving SNAP benefits. However, 8 (67%) participants answered yes to having ever received SNAP benefits. In comparison, the majority of participants (9/12, 75%) scored low or very low for food security according to the 6-Item Food Security Scale [38].

All participants used smartphones regularly to access the internet, and nearly all (10/12, 83%) also used a desktop computer or laptop. During usability testing, 5 participants chose to test the web-based tool using an iPad, 4 used a laptop, and 3 used their smartphone. Participants used the same type of device for both round 1 and round 2.

### Usability Testing

#### Round 1

The most common positive and negative feedback for each category that emerged from the content analysis is presented in Tables 2 and 3. While participants generally conveyed that they had overall positive experiences with the tool, participants also provided various suggestions to improve the functionality of the website. Most concerns were related to visual design, navigation, home page, and the screening tool component.

**Table 2:** Summary of positive responses to round 1 version tool (N=12).

Response				Values, n (%)
**Positive feedback**				
	**Visual design and format**			
		Visual format is organized, easy to follow	5 (42)	
		Color scheme is attractive	3 (25)	
		Images of students reflect the diversity of the student body	3 (25)	
		Font is legible	3 (25)	
		Screener button stands out	3 (25)	
	**Home page**			
		Images of food items represent what can be purchased with SNAP^a^ benefits	6 (50)	
		Messaging and testimonials are relatable	3 (25)	
		Messaging and video are brief	3 (25)	
	**SNAP resources**			
		Listed resources are helpful and directly related to applying for SNAP	6 (50)	
	**FAQs^b^**			
		Listed questions are relevant, helpful, and easy to understand	8 (67)	
	**Other food resources**			
		Alternative resources to SNAP for food assistance are helpful	4 (33)	
	**Screening tool**			
		Light or dark mode toggle feature is useful	4 (33)	
		Feature to email results is useful	4 (33)	
		Explanations of questions are adequate	3 (25)	
	**Other**			
		Tool is easier to use compared to government websites	2 (17)	
		Website conveys an encouraging message that SNAP could help	2 (17)	
		Website is a good first step to learning about SNAP	2 (17)	

^a^SNAP: Supplemental Nutrition Assistance Program.

^b^FAQ: frequently asked question.

**Table 3:** Summary of negative responses to round 1 version tool (N=12).

Response			Values, n (%)
**Negative feedback**			
	**Visual design and format**		
		Website logo does not look good	3 (25)
		Images, textboxes, and buttons are not formatted correctly for the user’s device	3 (25)
		Visual identity is ambiguous and lacks credibility	2 (17)
	**Navigation**		
		No clear way to navigate to the home page	6 (50)
		Did not realize that there are informational sections other than the screening tool	5 (42)
		Did not realize that the home page contains the information below the header section	5 (42)
	**Home page**		
		Images of students should be more relatable	2 (17)
		Testimonials should be more relatable	2 (17)
		Animation of images is confusing	2 (17)
	**SNAP^a^ resources**		
		More information about the SNAP application process (eg, documents needed and timeline) should be included	2 (17)
	**Screening tool**		
		Monthly income questions are confusing and difficult	6 (50)
		Citizenship and immigration question page is confusing	5 (42)
		Toggle-like buttons for yes or no questions are unintuitive to use	4 (33)
	**Other**		
		Hyperlinks should open in a new tab rather than in the same tab	3 (25)
		Definition and more information about SNAP should be included	2 (17)

^a^SNAP: Supplemental Nutrition Assistance Program.

Three participants conveyed dislike for the website’s logo, which initially only included text stating “SNAP-FOR-U,” and 3 participants also noted poor formatting of images, textboxes, and buttons that did not fit properly on the screens of their mobile devices. As an extension, 2 participants stated they found the visual identity of the tool to be ambiguous, leading to a perceived lack of credibility. Visibility concerns were also raised as a few participants encountered parts of the website having white-colored text over a white background. Students recommended adding a more visual element to the logo and providing information about who is responsible for the tool to ensure that users can trust the information provided by the website.

Navigation was a major concern for around half of the participants. Upon initially accessing the website, participants reported that they did not realize the tool was composed of informational sections other than the screening tool. Additionally, participants had difficulty navigating back to the home page due to a lack of a discrete “home page” button aside from the website logo. Some stated it felt like “a guessing game” to see if the website logo would bring the user back to the home page or not. Participants suggested clearer navigation symbols and features to allow users to know about and access different parts of the website more easily.

Although experiences with the home page were mostly favorable after participants were informed about additional content below the header image, some participants expressed wanting a more personal and relatable experience. Participants suggested that the images, messaging, and testimonials should directly reflect the institution they attended, rather than using stock images or testimonials from students attending other institutions. Additionally, while the imagery of food items was a helpful visual to half of the participants, a few found the automatic movement of the images to be frustrating, especially when they wanted to manually go back to a picture they missed.

The screening tool was another aspect where many participants encountered confusion and difficulty. For ease of answering the survey, many questions were designed to be answered as binary yes or no questions that featured a toggle-like button. However, participants found it unintuitive to answer these questions as the button responded from single taps instead of sliding movements as most participants initially thought. Income questions were difficult for several participants for reasons including the lack of clarity on whether the questions should be answered based on the individual or household, how to calculate their income, and just not knowing about their household’s income. Suggestions to overcome these issues included providing instructions or calculations on how to derive this information or providing an indication that this information would be needed prior to starting the screening tool. The page containing a question on citizenship and immigration also caused confusion as it was a read-only page. However, participants did not realize this and felt frustrated when trying to interact with the question due to the lack of obvious indication or visual feedback from the tool to inform the user that the answer was not intended to be answered. Some participants reported they would have felt comfortable answering the question, while others recommended that the information be placed elsewhere to avoid the perceived risk of disclosing such personal information.

Other suggested improvements for the tool included having hyperlinks open in new browser tabs and providing more information related to SNAP and the SNAP application process. Participants sought information on how to apply for SNAP, especially if their screening results suggested they may be eligible and were disappointed in the lack of instruction and advice provided by the tool itself. Referrals to external sources such as government SNAP pages were insufficient. Participants also acknowledged that inconveniences or unintuitive features led to feelings of frustration, leading to questioning whether the user or the tool was at fault for the difficulties experienced. Overall, user experiences for round 1 were generally favorable, with most participants (10/12) stating that the website fulfills its primary objective as a support tool to encourage college students to apply for SNAP.

#### Website Changes Between Rounds

After the first round of usability testing, researchers discussed participants’ feedback to debrief the website developer. Comments from participants’ user experiences and context from voluntarily disclosed personal experiences with SNAP informed the modifications to create the second iteration of the tool (Multimedia Appendix 2). Major feedback included comments about unclear navigation regarding the home page, lack of clarity at specific parts of the screening tool, suggestions for more information about the SNAP application and eligibility criteria, typographical errors, and functionality improvements. Researchers reviewed the feedback and provided suggested changes to the developer to address the various concerns from the first round.

**Figure 2:**
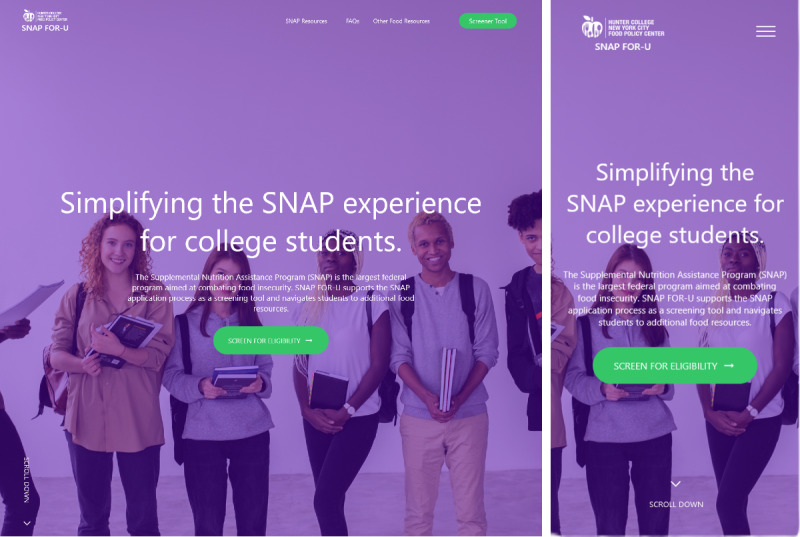
Round 2 home screen of the SNAP For-U webpage on desktop (left) and mobile devices (right).

Initially, many participants did not realize that the home page contained information below the home page image and screening tool button. For better clarity of navigation, a visual aid was added to encourage users to scroll and the navigation bar was updated (Figure 2). Participants also requested more information about how to apply for SNAP, so a step-by-step guide was added adjacent to already listed SNAP resources. Other significant changes made to the website include updated wording and descriptions in the screening tool to improve the clarity of questions. An embedded calculator feature was also added to the screening tool to address difficulties in answering income-related questions. Initially, participants expressed disappointment in the lack of transparency to explain how the screener produced the results. The conditional branching of the screening tool was updated along with the results page to provide a specific rationale for ineligible results based on input to the screening tool and general SNAP eligibility information. Other changes were made to improve functionality and responsivity of interactive features, creating a better user experience.

#### Round 2

After implementing a number of changes to reflect round 1 feedback, participants returned to test the second version of the web-based tool using the same devices they used in round 1. The most common feedback is summarized in Tables 4 and 5. Similar to round 1, most participants expressed positive user experiences, with 4 conveying that the website now felt “complete” or “polished.”

In this second round of testing, 7 participants noted the change to the website’s logo and expressed approval for the change. Participants also noted the updated navigation bar. However, a few recommendations were made to further provide clarity and improve the website’s navigation. Two participants reiterated their recommendation for a separate home page button on the navigation bar or menu list in addition to the website’s logo. An additional recommendation included a better visual indication of the user’s current page from the menu list by highlighting the section they were in.

Changes to the home page were also well received. While most participants liked the change to the movement of the food items images, a few noted that the directional indicators were confusing as they did not match the actual movement of the images. Students expressed a preference for left and right arrows to assist with navigating the images, in addition to the ability to drag the images leftwards and rightwards. Three students, like in round 1, stated they would prefer to have more relatable images and testimonials from students who attend the same institution as them. Two students suggested including information about SNAP’s eligibility requirements on the home page as this would be helpful to students who may not want to complete the screener.

The addition of the step-by-step SNAP application guide was found to be helpful, but most participants initially overlooked the inclusion. Some reported that the guide did not stand out against the other resources included on the page and suggested larger fonts or changing font colors for visual indication.

The frequently asked questions continued to be a highly approved section, but students recommended improving the organization by reordering or grouping questions by topics so that logically connected questions would be next to each other. A few students also noted that hyperlinking relevant resources within certain questions would be helpful by minimizing the amount of work a user would need to do to access the resources or documents mentioned.

Despite significant changes to the screening tool, 5 participants continued to experience difficulty with income questions due to a lack of awareness of their own household’s income. They suggested that a disclaimer be provided at the beginning of the screening tool so that users would know to have the information ahead of time. Participants who found the calculator feature on their own generally liked it, but several did not realize that a calculator feature was available due to the calculator blending in with the question. These users suggested that the calculator be placed above the answer input box for the related questions. While participants approved the additional information and explanation provided by the results page, 2 participants continued to find the information on the “ineligible” results page to be insufficient. They wanted more detailed information about eligibility criteria and how they could change from being likely ineligible to eligible. In the context of overall user experiences, almost all participants (11/12) thought that the website is an effective support tool to encourage SNAP enrollment in college students.

In addition to feedback for immediate modifications or additions to the web-based tool, participants also shared considerations for future implementations and for expansion of initiatives around supporting SNAP enrollment in college students. A few students expressed that translating the tool to different languages would be useful as they perceive that a significant portion of urban college students are not native English speakers and may not be sufficiently fluent to navigate the SNAP application process, or may have family members who are not literate in English. Participants reasoned that engagement with the website would increase as users would feel more confident interacting with content in a familiar language. Other participants, while acknowledging that this tool is “a good first step” to the SNAP process, conveyed that the tool’s impact may be limited if students are unable to access individualized assistance, such as a social worker or coordinator, when they attempt to apply for SNAP. It was suggested that the tool could also serve to direct students to in-person assistance at their school or within their locality to help students navigate government bureaucracy.

**Table 4:** Summary of positive responses to round 2 version tool (N=12).

Response			Values, n (%)
**Positive feedback**			
	**Visual design and format**		
		Website logo looks good	7 (58)
		Website is legible, well organized, and easy to follow	6 (50)
		Color scheme is attractive	4 (33)
	**Navigation**		
		Menu icon is good	2 (17)
	**Home page**		
		Scroll down” visual to be a helpful indicator	8 (67)
		Highlighted key phrases in messages are good	4 (33)
		Information about SNAP^a^ retailers is useful	4 (33)
	**SNAP^a^ resources**		
		Information in the step-by-step guide is useful	4 (33)
	**FAQs^b^**		
		Listed questions are relevant, helpful, and easy to understand	5 (42)
	**Other food resources**		
		Alternative resources to SNAP for food assistance are helpful	6 (50)
	**Screening tool**		
		Questions were overall easy to answer and the results page was easy to understand	4 (33)
		Calculator feature is helpful	2 (17)
		Screener result is accurate to the participant’s experience	2 (17)
	**Other**		
		Overall website feels complete and informative	4 (33)

^a^SNAP: Supplemental Nutrition Assistance Program.

^b^FAQ: frequently asked question.

**Table 5:** Summary of negative responses to round 2 version tool (N=12).

Response			Values, n (%)
**Negative feedback**			
	**Navigation**		
		Menu sections should be highlighted based on the user’s current page	2 (17)
		Discrete “home” button should be on the navigation bar	2 (17)
		Hyperlinks at the bottom of the website should bring the user to the top of the newly loaded page	2 (17)
	**Home page**		
		Arrows next to images of food items should point left and right, instead of upwards	3 (25)
		Testimonials and images of students should be more relatable	2 (17)
		Information about SNAP eligibility requirements should be included	2 (17)
	**SNAP^a^ resources**		
		Visual differentiation of the step-by-step guide should be improved	6 (50)
	**FAQs^b^**		
		Hyperlinks should be included in questions that mention a resource	3 (25)
		Ordering or grouping questions should be improved	2 (17)
	**Other food resources**		
		Text of headings run outside the textbox	3 (25)
	**Screening tool**		
		Monthly income questions are difficult	5 (42)
		Calculator feature is not apparent	4 (33)
		Information about SNAP eligibility requirements should be included at the beginning	2 (17)
		More information on what to do if the user receives an “ineligible” result should be included	2 (17)
	**Other**		
		Some hyperlinks do not open in new tabs	3 (25)

^a^SNAP: Supplemental Nutrition Assistance Program.

^b^FAQ: frequently asked question.

### SUS

Overall, SUS scores in both round 1 and round 2 (median 91.25, IQR 82.50-95; median 92.50, IQR 81.25-96.09, respectively) indicate high usability. When compared to acceptability ranges as presented by Bangor et al [39] (below 50 is unacceptable, 50 to 70 is marginally acceptable, and over 70 is acceptable), the overall SUS scores in both rounds indicate that this tool is acceptable in usability. In comparison to a school grading scale, the scores from both rounds can be interpreted as a B for scores between 80 and 89 and an A for scores 90 and 100 [39]. Participants’ responses to their likelihood to recommend the tool were found to be high in both round 1 and round 2 (mean 8.83, SD 2.1; mean 9.33, SD 1.4, respectively; *P*=.25), indicating good acceptability of the tool.

## Discussion

### Principal Findings

The objective of this study was to evaluate the perceived usability and acceptability of an informative web-based tool prototype to encourage SNAP enrollment among urban college students. Through 2 rounds of usability testing, the evaluation of the tool generated overall positive user experiences and further considerations to modify and add to the tool to improve usability and increase potential engagement and impact.

Determining effective modalities to address food insecurity in younger disadvantaged populations, such as college students, continues to be a public health concern. Literature indicates that younger populations increasingly look toward the internet as a trusted resource for health information compared to traditional media [40,41]. However, common difficulties associated with accessing health-related information include determining reliability and the ability to find information of interest [42]. Past studies have also identified that key barriers to college students accessing food assistance programs, such as food pantries or governmental benefit programs, include social stigma, unclear program information, and difficult application processes or other administrative burdens [2,12,43,44]. This study applied a college student–centric approach to create an informative web-based tool, which could alleviate obstacles that food-insecure students face when applying for SNAP. The features and design of SNAP For-U reflect previous findings in which students have suggested the need for greater awareness, positive messaging, and access to information, along with preferences for resources that appear credible, contain quality information, and are easy to use [42,43,45]. Creating a web-based tool that fulfills the information-seeking criteria sought by young adult audiences can increase engagement, leading to increased self-efficacy in facilitating learning and modulating behavior intentions.

In both rounds, we observed high usability and acceptability through user interactions, verbal feedback, and the SUS questionnaire. Participants consistently expressed approval for the concise wording and organization of the tool’s informational sections. The inclusion of multiple methods to access the screening tool throughout the website was effective in directing participants to the screening tool, the main component within the overall tool. Although it is suggested that redundancy in website design can negatively increase cognitive load for users, participants expressed that some redundancy provided the benefits of better visibility for important features and key information [46,47]. This complements previous research that found that redundant user interface design improves speed and accuracy in using the product for new users with moderate to high familiarity with technology [48]. A few participants compared the visual and informational presentation of this study’s tool to government-affiliated websites or other similar websites, conveying that the tool’s streamlined format with attractive imagery was preferable, whereas government-affiliated websites can sometimes feel “overwhelming.” This is consistent with findings from Lee et al [49], where the textual presentation of information on government websites was associated with higher information overload, which had a negative relationship with perceived usefulness [49]. Furthermore, evidence also suggests a direct relationship exists between perceived usefulness or usability and trust in government websites [50,51].

While some participants felt that the tool offered sufficient information in round 1, others recommended adding more information to provide a complete guide to the process. Most participants also experienced difficulties with navigation and difficulties with the screening tool. Adding an application guide, improving navigation features, and updating the screening tool with clarified wording and an added calculator tool aimed to address these issues. In round 2, all participants were able to navigate through the website with ease, with only a few participants providing additional minor considerations to further improve the clarity of navigation. While a discrete “home page” button was not added for round 2, probing revealed that most participants felt that it is common for website logos to also function as a home page button. However, participants still preferred having an additional button stating “home” or “home page” to eliminate the need to guess. After testing in round 2, when asked, most users expressed that the tool was sufficient in providing a thorough and accurate overview of the SNAP application process. However, some participants mentioned that presenting key information, such as eligibility criteria, in multiple locations would provide better visibility and emphasize the most important information about SNAP. The questions in the screening tool were noted to be comparable to those of the actual SNAP applications. Moreover, the resulting estimated benefit amount was perceived as particularly encouraging as it provided a quantified expectation. Usability testing underscored the importance of providing adequate instruction to decrease the effort needed to learn and effectively use the web-based tool. Participants often preferred specific and detailed but concisely worded information instead of generalized information to better address the varying knowledge levels between different users. Appealing visual design was also an important aspect that mediated the perception of credibility, usability, and usefulness. As usability scores were high to begin with, differences between round 1 and round 2 usability scores were not statistically significant. However, participants’ feedback on the revisions further reaffirmed positive perceptions of the tool as appealing, easy to use, and effectively providing support for the SNAP application process.

Overall findings reinforce the value of usability testing with representative end users. Despite tool development being informed by user-centered design principles and iterative testing within the research team, study participants highlighted several issues and presented various future considerations that did not emerge during internal testing. Of note, sections of the SNAP screening tool had to be revised in consideration of the perceived difficulty of questions and the limited baseline SNAP knowledge of potential end users. Through a combination of qualitative and quantitative methodology to conduct usability testing, a number of usability issues along with potential user-suggested solutions were able to be discovered.

### Limitations

Although this mixed methods approach to usability testing produced extensive and valuable insights from student testers to inform necessary tool improvements, this study is not without limitations. While the study sample size is typical for usability testing, our results may not be generalizable to the overall target audience of the tool and almost all participants self-identified as female [16-21]. This may have led to the underrepresentation of nuances in web-related user experiences from perspectives of other genders.

A common limitation of think-aloud procedures is the risk of social acceptability bias due to the presence of 2 researchers during usability testing. Participants may have felt compelled to verbalize positive feedback or may have interacted with the tool differently than in a natural setting. Another consideration is that participants may have perceived the round 2 prototype as more usable or easier to learn due to their previous experience during round 1. However, maintaining the same participants for both rounds was necessary to make direct comparisons of both iterations of the tool. Finally, the mixed modalities of participation may have contributed to varying amounts and qualities of information captured in each usability testing session. In-person sessions allowed for better documentation of a participant’s body language. Conversely, remote sessions enabled researchers to observe the entirety of a participant’s interactions with the tool through screen sharing.

### Future Research

The findings from this study will inform further development of this tool. However, the tool’s usability and acceptability should also be tested with other cohorts of college students who may be at increased food insecurity risk and pilot-tested to determine potential effectiveness. Future expansions of this tool may include considerations for language and other accessibility features, additional connected support resources, and modifications for other at-risk populations or public assistance programs, such as young pregnant women and Special Supplemental Nutrition Program for Women, Infants, and Children. Our study also provides broader guidance for web-based tool development of other similar public health-orientated interventions for young and technologically proficient audiences.

### Conclusions

Usability testing is vital to the development of web-based tools to determine the usefulness of a tool and to gain insights to further enhance its effectiveness, usability, and engagement among its end users. The researchers engaged representative student participants using a standard combined think-aloud approach, gaining immediate impressions during the testing of the tool and retrospective feedback contextualized by the users’ overall experiences. These perspectives will help to refine the tool in its next iterations. Overall usability findings suggest that this web-based tool was highly usable and acceptable among urban public college students and thus could be an effective and appealing approach as a support tool to introduce college students to the SNAP application process. This tool could eventually be disseminated across various urban college campuses and adapted for other localities or nonurban regions to encourage SNAP use among college students.

## Appendix

Multimedia Appendix 1 Screenshots of SNAP For-U V1.

Multimedia Appendix 2 Screenshots of SNAP For-U V2.

## Abbreviations

REAM: Rapid Evaluation and Assessments Methods
RITA: Rapid Identification of Themes From Audio Recordings
SNAP: Supplemental Nutrition Assistance Program
SUS: System Usability Scale

## Data Availability

The data sets generated and analyzed during this study are available from the corresponding author on reasonable request.
