# Supplementary material for: Web-Based Tool Designed to Encourage Supplemental Nutrition Assistance Program Use in Urban College Students: Usability Testing Study
Source: JMIR Form Res. 2024 Jun 13;8:e50557. doi: 10.2196/50557 (PMC11211703; doi:10.2196/50557)
Supplement: Multimedia Appendix 1 [file formative_v8i1e50557_app1.pdf]

SNAP FOR-U

SNAP Resources | FAQs | Other Food Resources

Screener Tool

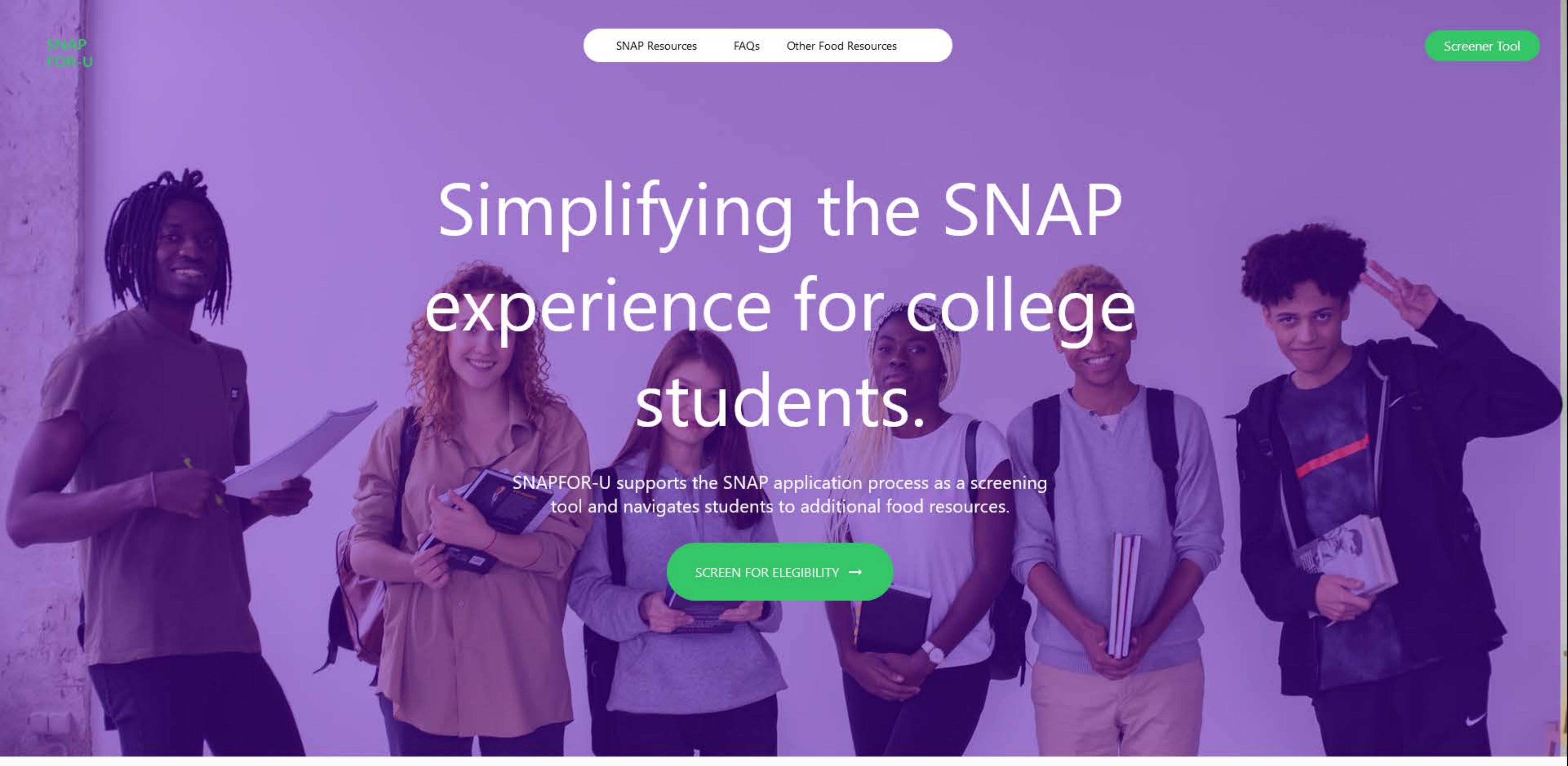

Simplifying the SNAP experience for college students.

SNAPFOR-U supports the SNAP application process as a screening tool and navigates students to additional food resources.

SCREEN FOR ELEGIBILITY →

Access to food is a right, not a privelege.

Everyone deserves to eat! SNAP is an entitlement program, which means there's always assistance available for eligible applicants. If you're eligible, you'll get SNAP benefits **within 30 days** of applying!

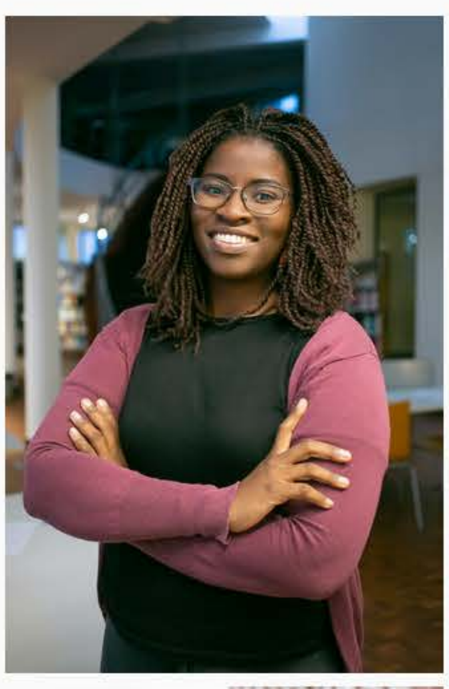

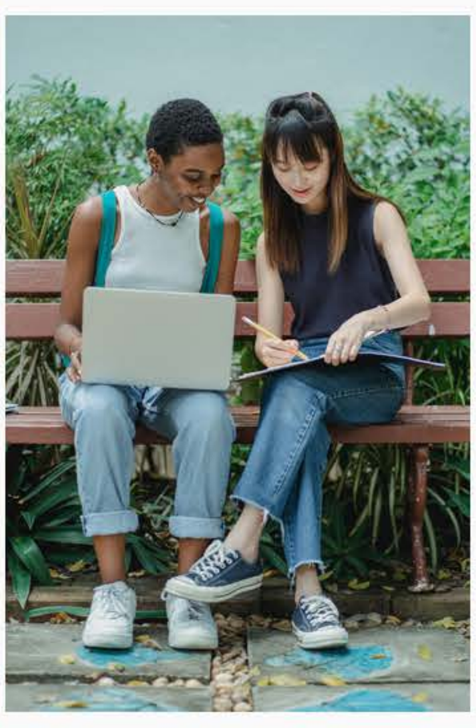

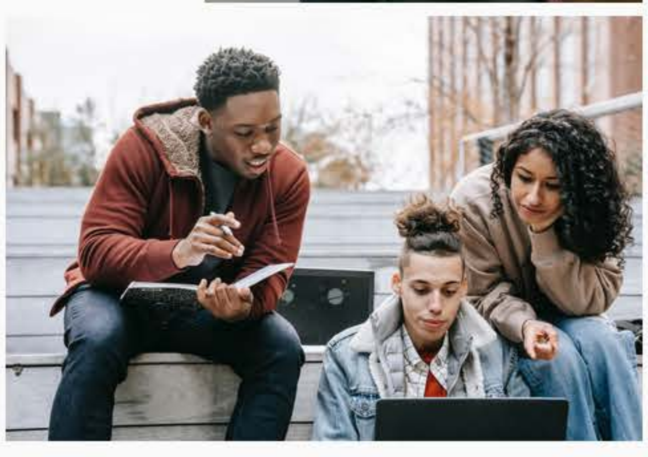

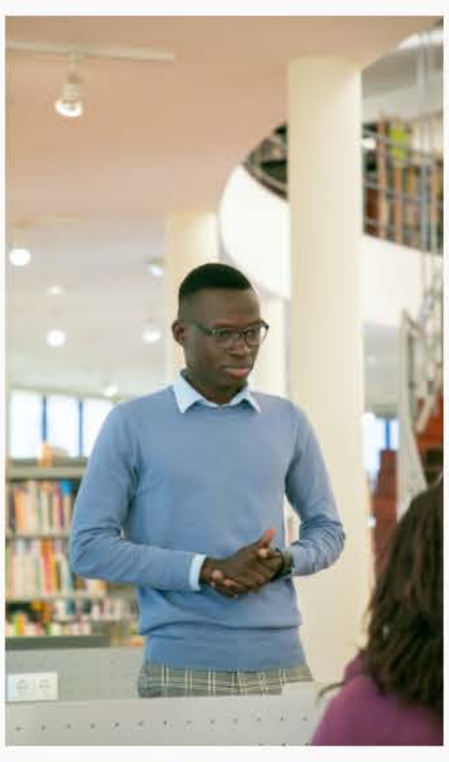

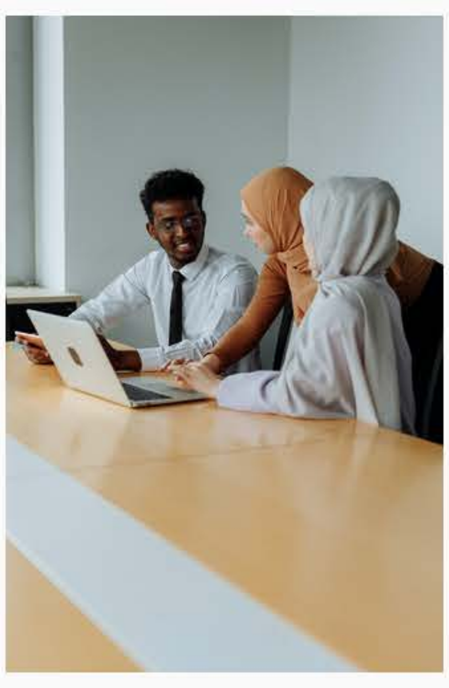

No worries! SNAP has got you covered.

Over **2 million students** across the U.S. are eligible for SNAP. Did you know that in NYC **only half** of eligible 18 to 29 year olds are receiving SNAP benefits?

A SNAP recipient receives an average of **$175-181** in benefits each month. You could be one of them!

NOT SURE IF SNAP IS FOR YOU?

Hear what others have to say about SNAP.

S

- SARAH, DUKE UNIVERSITY GRADUATE

"I felt really bad about being on food stamps for a while. 'They're just for people who really really need it,' I thought... Eventually I sort of realized that even though I am 24, I still have responsibilities. I still have hopes for my future, and if I need to be on food stamps to make that happen, then maybe I do really need it."

- KARIMA, CITY

"SNAP as a program is great.
you n

← →

SNAP Made Easy.

Share

Here's how you can use SNAP.

SNAP benefits are provided through an Electronic Benefit Transfer (EBT) card that you can use just like any credit or debit card. The benefits can be used at grocery stores, farmers markets, bodegas, delis and for online grocery purchases.

Check which retailers accept SNAP payments.

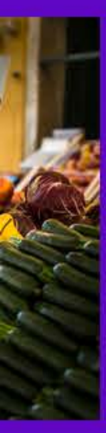

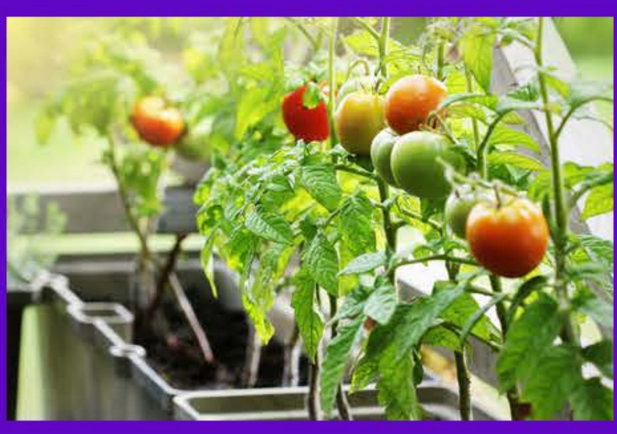

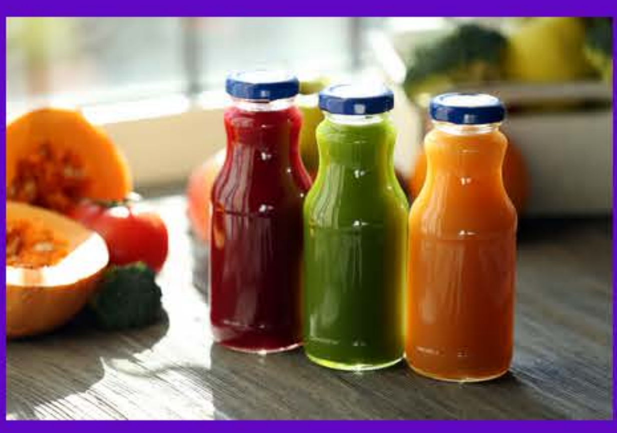

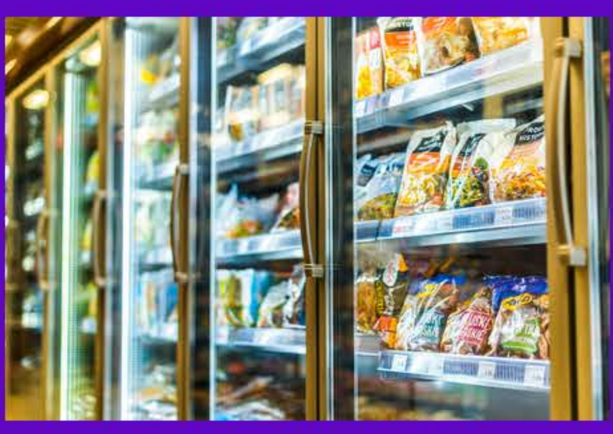

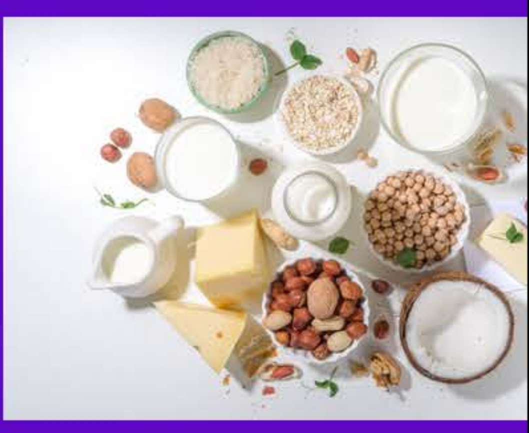

CLICK CARD TO REVEAL MORE INFORMATION

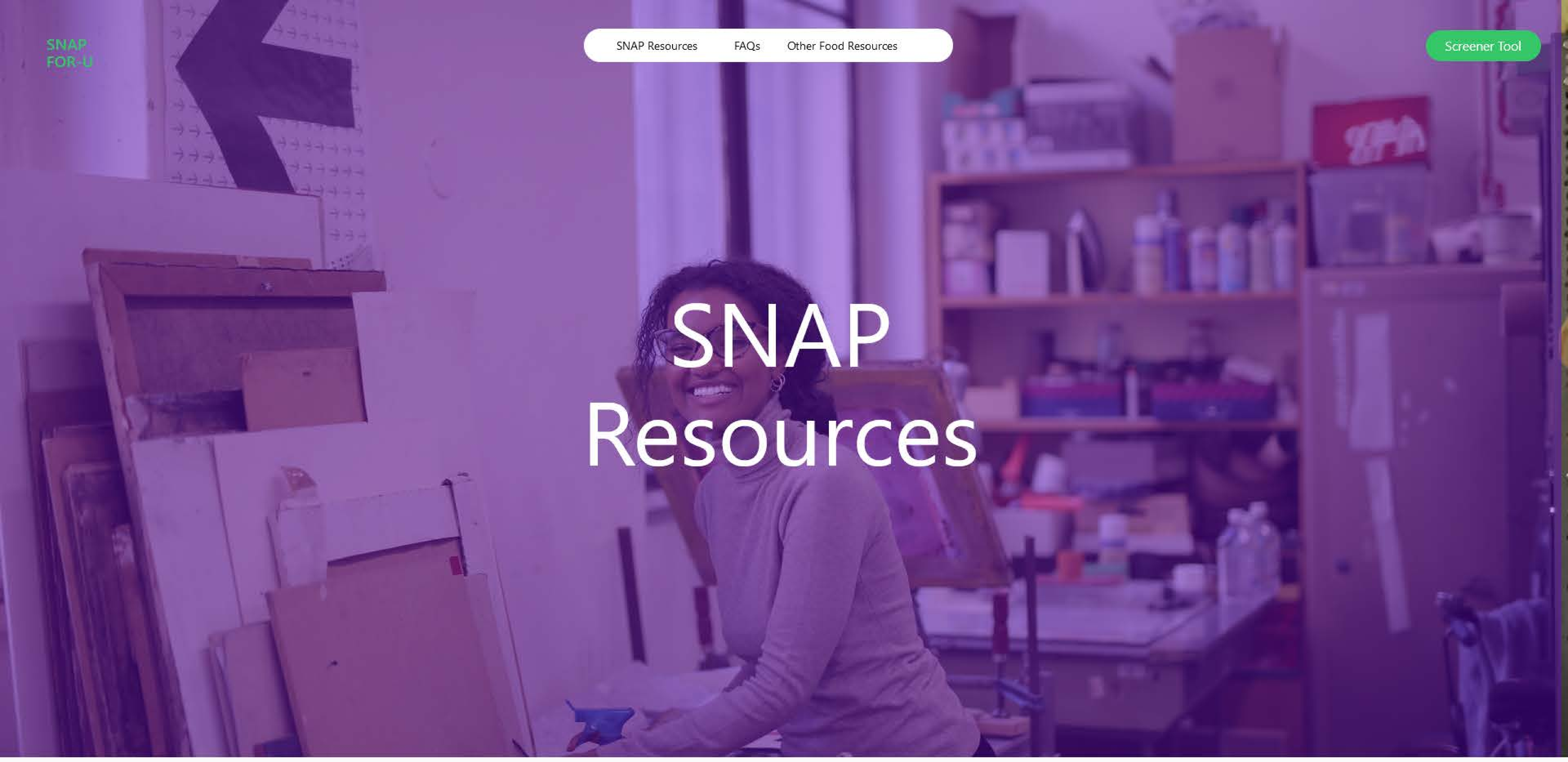

SNAP Resources

SNAP Resources Guide

Looking to apply for SNAP benefits?

These resources can help guide your next steps.

Access HRA

With ACCESS HRA, you can apply for some HRA benefits, submit documents for your application, manage your case, and much more!"

Visit Resource

SNAP Application/Recertification Form

This is a digital pdf version of the SNAP Application/Recertification Form.

Visit Resource

SNAP Centers

This is a map and list of Supplemental Nutrition Assistance Program (SNAP) center locations.

Visit Resource

SNAP Documentation Guide (COVID-19)

This guide provides a list of suggested documentation to provide with your application to determine your SNAP eligibility and benefit amount.

Visit Resource

For CUNY students

CUNY SNAP page

This is CUNY's guide to applying for SNAP. You can access information on how to obtain a "Student Verification of Enrollment" on CUNYfirst to provide as documentation with your application.

Visit Resource

CUNY Food Navigator

This is a free digital referral service for any CUNY student who is looking for help with accessing food and other basic necessities needed to get through these challenging times.

Visit Resource

Disclaimer

All estimates and data on this website are for educational purposes only and provide a basic estimate based on publicly available information. State test, benefit, and allowance amounts may be incomplete, incorrect, or outdated due to limitations in finding updated sources. Eligibility factors vary and eligibility criteria will not be included here. The only way to find out your true eligibility and benefit amount is to apply. This website is not affiliated with any government organization or SNAP program.

@2022 HUNTER COLLEGE

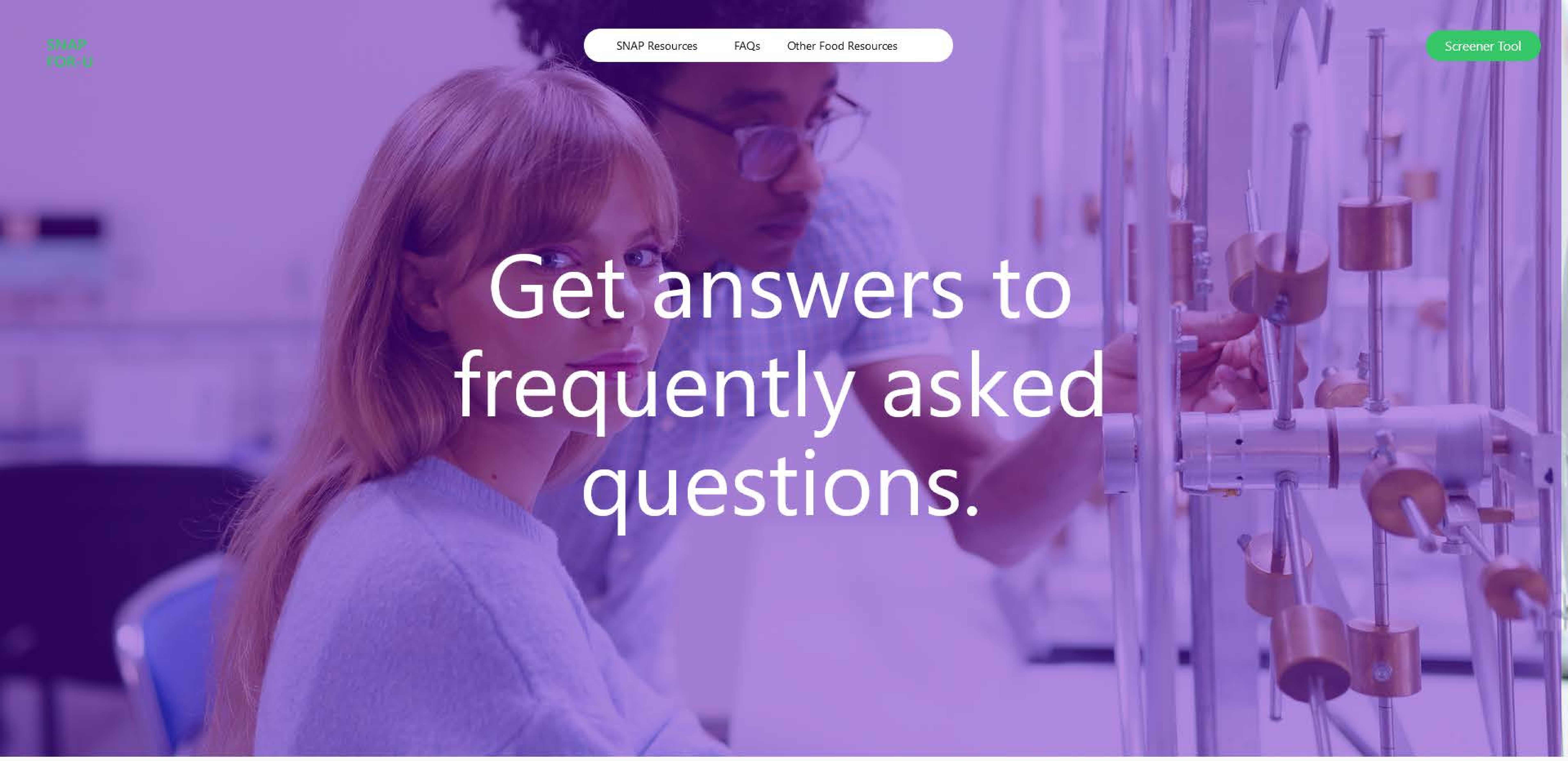

Get answers to frequently asked questions.

Support

FAQs

Everything you need to know about SNAP assistance. Can't find your answer here? Check out more FAQs **here**

What is SNAP?

The Supplemental Nutrition Assistance Program (SNAP), formerly called Food Stamps, is the largest federal program aimed at combating hunger and food insecurity. SNAP benefits are provided through an Electronic Benefit Transfer (EBT) card, similar to a bank debit card or credit card.

Do I need to meet the required number of work hours?

No. There are other exemption criteria that college students can qualify under instead of working an average of 20 hours per week.

Do I need to have a Social Security Number (SSN) to be eligible for benefits?

What is a "SNAP household"?

I am financially dependent on my parents. Can I still apply for SNAP benefits?

I am not the head of my household, but can I apply for SNAP and attend the interview on their behalf?

Does financial aid count as income?

I am attending school in a state different from the one of my official residence. In which state should I apply for SNAP benefits?

Will receiving SNAP benefits affect my immigration status?

Can I check my application's status online?

My parents don't speak English well. Is there any way to get interpreter services to help them apply for SNAP?

Do I meet the required number of work hours?

Do I need to apply in person?

Disclaimer

All estimates and data on this website are for educational purposes only and provide a basic estimate based on publicly available information. State test, benefit, and allowance amounts may be incomplete, incorrect, or outdated due to limitations in finding updated sources. Eligibility factors vary and eligibility criteria will not be included here. The only way to find out your true eligibility and benefit amount is to apply. This website is not affiliated with any government organization or SNAP program.

@2022 HUNTER COLLEGE

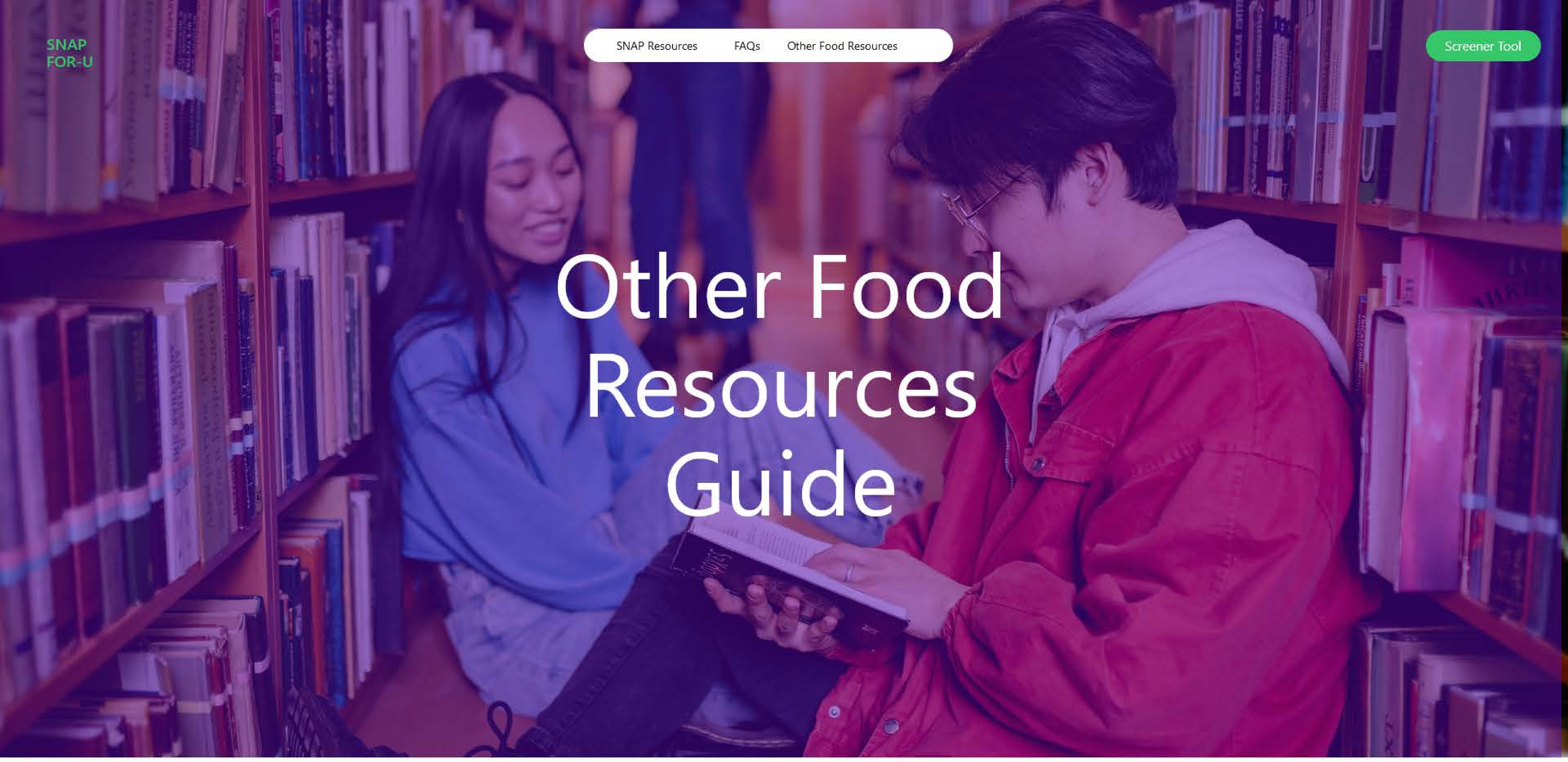

Other Food Resources Guide

Food Resources Guide

Check out the collection of food resources below.

Our top recommendations for food resources within the community.

NYC Neighbourhood Food Resource Guides

To help connect community members in need with food resources, the Hunter College NYC Food Policy Center created NYC Neighborhood Food Resource Guides for each NYC neighborhood.

Visit Resource

Share Meals

Share Meals is dedicated to ensuring all college students are food secure. We empower college students to strengthen their own communities through sharing food and we accomplish this through technology, activism, and advocacy.

Visit Resource

Food Help NYC

Food Help NYC is an application that can be used to find locations that offer free food (food pantries and soup kitchens). It discloses the hours opened and address of each location, however, these may be subject to change.

Visit Resource

Too Good To Go

Our mission? To make sure good food gets eaten, not wasted. Every day, delicious, fresh food goes to waste at cafés, restaurants, hotels, shops and manufacturers - just because it hasn't sold in time.

Visit Resource

Health CUNY - Food Pantries

This is a list of all food pantries at CUNY campuses.

Visit Resource

Disclaimer

All estimates and data on this website are for educational purposes only and provide a basic estimate based on publicly available information. State test, benefit, and allowance amounts may be incomplete, incorrect, or outdated due to limitations in finding updated sources. Eligibility factors vary and eligibility criteria will not be included here. The only way to find out your true eligibility and benefit amount is to apply. This website is not affiliated with any government organization or SNAP program.

@2022 HUNTER COLLEGE

SNAP
FOR-U

Welcome to SNAP FOR-U!

This screener tool is designed to help you determine your potential eligibility for SNAP benefits and estimate the amount you could receive.

Please note this is an unofficial tool and is not affiliated with any government organization. This screener only provides a general estimation based on public information as of August 2022.

At the beginning of the screening questions you can choose to save your progress so that you may stop at any time and continue at a later time. You can also go back to any question and freely edit your answers. You will also be able to review your answers before submitting. After completing the screening, you will be given a choice to email the results for your records or to use as a "cheat sheet" to help you fill out your SNAP application.

GET STARTED →

Disclosure of Privacy

This website will automatically collect information about how visitors use the website. (e.g. page views, traffic, error messages).

We would also like to collect some demographic data to improve this website. Collected information will include submitted answers to the screening tool questions. None of the questions ask for any personal identifiers. The data will not be used to identify any individual and will only be used to improve the screening tool and the other features our website offers.

- [ ] I consent to the collection of my data
- [ ] I do not consent to the collection of my data

GET STARTED →

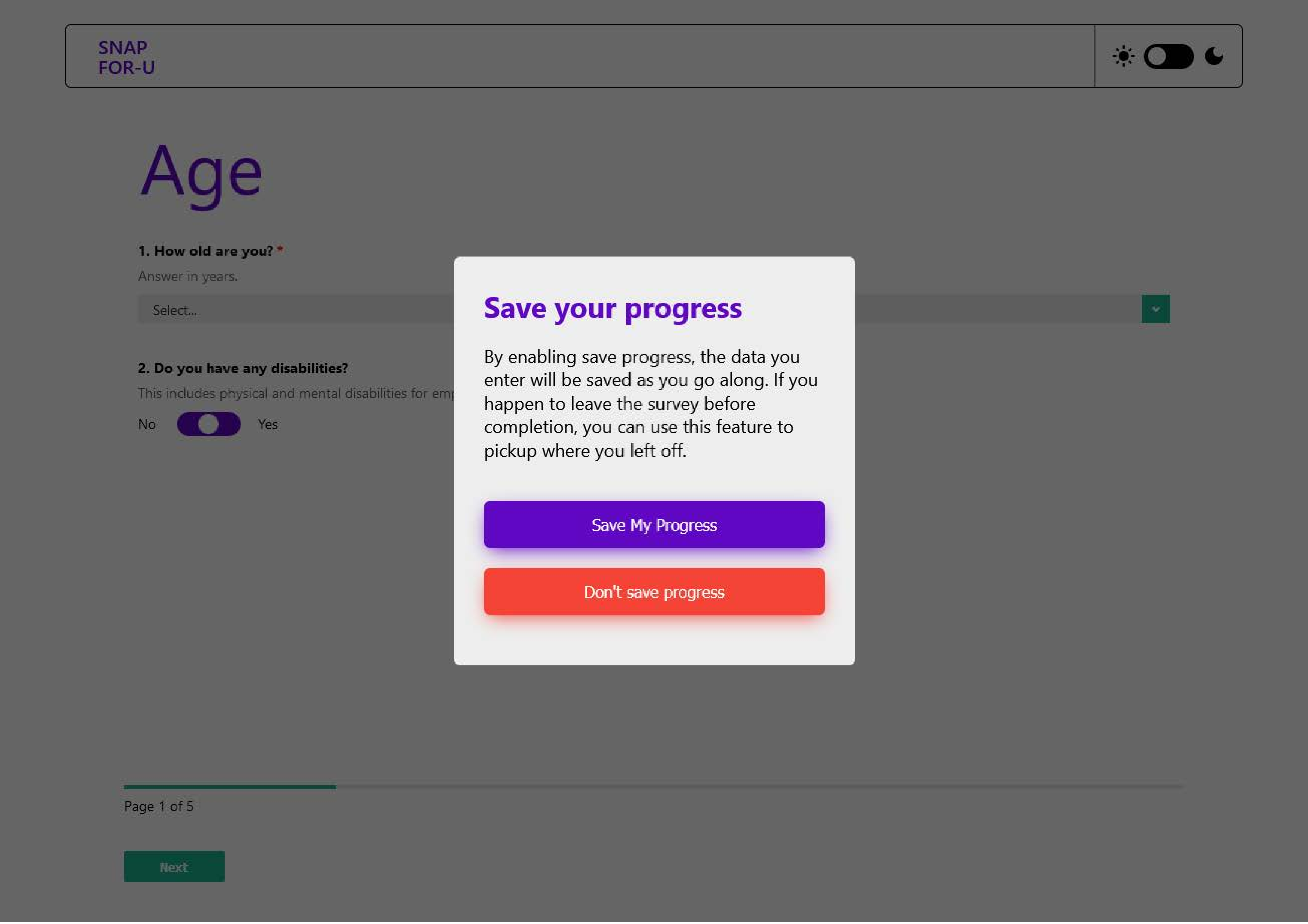
SNAP
FOR-U
Age
1. How old are you? *
Answer in years.
Select...
2. Do you have any disabilities?
This includes physical and mental disabilities for emp
No
Yes
Save your progress
By enabling save progress, the data you enter will be saved as you go along. If you happen to leave the survey before completion, you can use this feature to pickup where you left off.
Save My Progress
Don't save progress
Page 1 of 5
Next

SNAP
FOR-U

Student Status

3. Are you enrolled in a higher education institution or intend to enroll next semester? *

Includes but not limited to: colleges, universities, online courses, vocational/trade/technical schools, any education institution at the post-high school level

No Yes

4. What is your enrollment status? *

This is defined by your academic institution. Typically, "at least half-time" is considered to be 6 credits or more.

- (●) At least half-time
- ( ) Less than half-time

5. Are you enrolled in a college meal plan? *

No Yes

Page 2 of 7

Previous Next

SNAP
FOR-U

Student Exemptions

6. Which of the following criteria best fits you? *

Select ALL that apply. Choices denoted by '*' are temporary exemptions that will remain in effect until COVID-19 is no longer declared a public health emergency.

- [ ] Work an average of 20 hours per week.
- [ ] Have an expected family contribution (EFC) of $0 in the current academic year under federal student financial aid rules.*
- [ ] Eligible to participate in state or federal work-study.*
- [ ] Participate in state or federal work-study.
- [ ] Attend a State University of New York (SUNY) or City University of New York (CUNY) community, comprehensive, or technology college and is enrolled in a qualified certificate or degree Career and Technical Education (CTE) program.
- [ ] None of the above

7. Check if your program of study qualifies as a CTE program

CUNY College

Type in your college's name

Remove

Add another program

Page 3 of 7

Previous

Next

Household

9. Who is part of your household? *

Household refers to a group of individuals who live together, AND typically purchase food and prepare meals together for home consumption.

- ◯ Just me
- ◯ More than one person

10. Are any of your household members elderly or disabled?

Elderly is defined as aged 60 years or above. "Disabilities" includes both physical and mental disabilities.

No Yes

11. Do you have dependent care costs?

(i.e. out-of-pocket cost for child care needed to maintain employment, attend vocational/employment training or pursue education)

No Yes

Income

12. Does your household have earned income? *

This refers to income from jobs or self-employment.

No Yes

13. What is your monthly unearned income?

This includes cash assistance programs, SSI, Social Security, veteran's benefits, unemployment, disability, child support, alimony, bank interest, and cash gifts.

14. Monthly Gross Income

0

Deductions/Expenses

15. Do you have any of the following expenses? *

Select all that apply.

- [ ] Rent or Mortgage
- [ ] Heating or cooling
- [ ] Utilities (e.g. electricity, gas, water, sewer)
- [ ] Telephone
- [ ] None

SNAP
FOR-U

Citizenship or Immigration Status

You do not have to answer this question.

16. In order to be eligible for SNAP each member of your household must fit within one of the following categories:

This is just for your information. You DO NOT answer this question.

- [ ] U.S. Citizen
- [ ] Lawful Permanent Resident under the age 18
- [ ] Lawful Permanent Resident in the U.S. for 5 years
- [ ] Lawful Permanent Resident with certain disability benefits
- [ ] Lawful Permanent Resident with 40 qualifying quarters
- [ ] Honorably discharged U.S. veteran, the spouse and unmarried dependent children
- [ ] Aliens on active duty in the U.S. military service, the spouse and unmarried dependent children
- [ ] Aliens paroled into the U.S. for at least one year
- [ ] Refugees, Cuban/Haitian Entrants, Asylees, Amerasian immigrants, individuals with deportation or removal withheld, Hmong or Highland Laotians, victims of human trafficking, parolees for at least one year, conditional entrants, North American Indians born in Canada and members of federally recognized tribes

Previous

Preview

SNAP
FOR-U

Age

1. How old are you? *

Answer in years.

23

2. Do you have any disabilities?

This includes physical and mental disabilities for employment purposes.

No Yes

Edit

Student Status

3. Are you enrolled in a higher education institution or intend to enroll next semester? *

Includes but not limited to: colleges, universities, online courses, vocational/trade/technical schools, any education institution at the post-high school level

No Yes

4. What is your enrollment status? *

This is defined by your academic institution. Typically, "at least half-time" is considered to be 6 credits or more.

- (•) At least half-time
- ( ) Less than half-time

5. Are you enrolled in a college meal plan? *

No Yes

Edit

SNAP
FOR-U

Thank you for completing the survey!

You may be eligible for SNAP benefits

Based on the information you have provided, you may be eligible to receive **$303.10** in SNAP benefits monthly.. However, this estimate is for educational purposes only. There are other factors considered to determine eligibility and benefit amounts on the actual SNAP application.

Interested in saving your answers for a later time?

Click the button below to submit a copy of your results to the email address enterred down below.

Enter your email address.

Send Results

Want to apply for SNAP?

Visit NYC's official SNAP information page.

Interested in other resources & guides?

Check out some other SNAP & food resource guides.

Retake Screener
