# Supplementary material for: Web-Based Tool Designed to Encourage Supplemental Nutrition Assistance Program Use in Urban College Students: Usability Testing Study
Source: JMIR Form Res. 2024 Jun 13;8:e50557. doi: 10.2196/50557 (PMC11211703; doi:10.2196/50557)
Supplement: Multimedia Appendix 2 [file formative_v8i1e50557_app2.pdf]

SNAP FOR-U

SNAP Resources | FAQs | Other Food Resources | Screener Tool

Simplifying the SNAP experience for college students.

The Supplemental Nutrition Assistance Program (SNAP) is the largest federal program aimed at combating food insecurity. SNAP FOR-U supports the SNAP application process as a screening tool and navigates students to additional food resources.

SCREEN FOR ELIGIBILITY →

SCROLL DOWN

Access to food is a right, not a privilege.

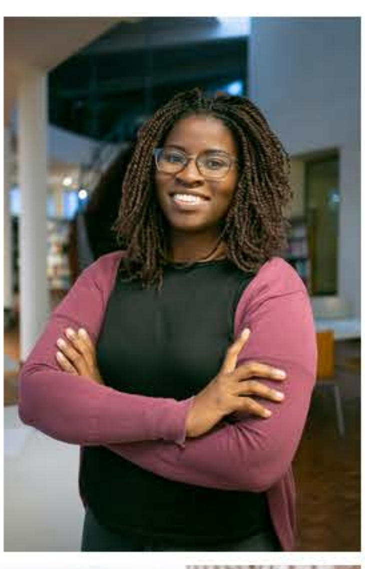

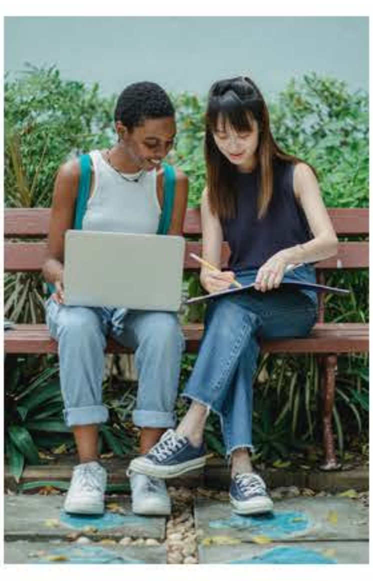

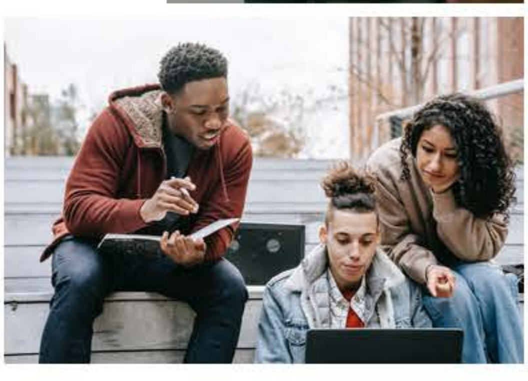

Everyone deserves to eat! SNAP is an entitlement program, which means there's always assistance available for eligible applicants. If you're eligible, you'll get SNAP benefits **within 30 days** of applying!

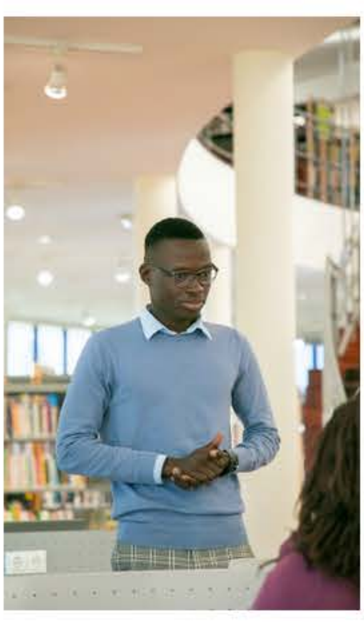

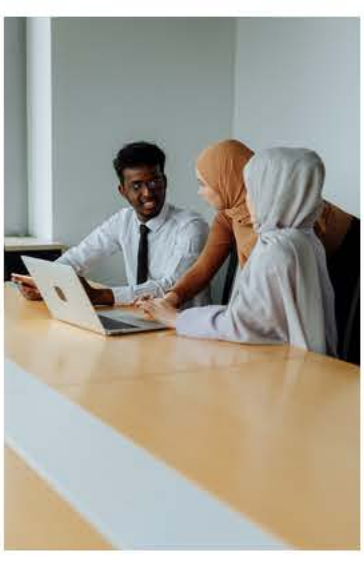

No worries! SNAP has got you covered.

Over **2 million students** across the U.S. are eligible for SNAP. Did you know that in NYC **only half** of eligible 18 to 29 year olds are receiving SNAP benefits?

A SNAP recipient receives an average of **$175-181** in benefits each month. You could be one of them!

NOT SURE IF SNAP IS FOR YOU?

See what others have to say about SNAP.

"I felt really bad about being on food stamps for a while. 'They're just for people who really really need it,' I thought... Eventually I sort of realized that even though I am 24, I still have responsibilities. I still have hopes for my future, and if I need to be on food stamps to make that happen, then maybe I do really need it."

- SARAH, DUKE UNIVERSITY GRADUATE

"SNA
oppo

- KA

SNAP Made Easy.

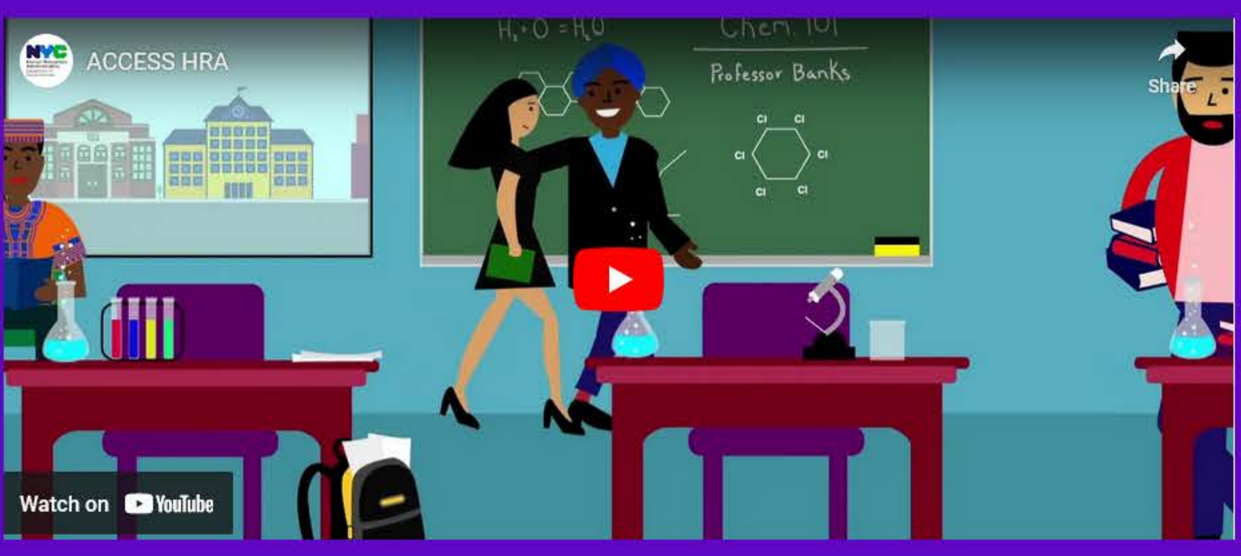

Here's how you can use SNAP.

SNAP benefits are provided through an Electronic Benefit Transfer (EBT) card that you can use just like any credit or debit card. The benefits can be used at grocery stores, farmers markets, bodegas, delis and for online grocery purchases. SNAP can be used to purchase most grocery items, but it can't be used to purchase hot prepared foods or food that can be eaten in the store.

Check which retailers accept SNAP payments.
Check which online retailers accept SNAP payments.

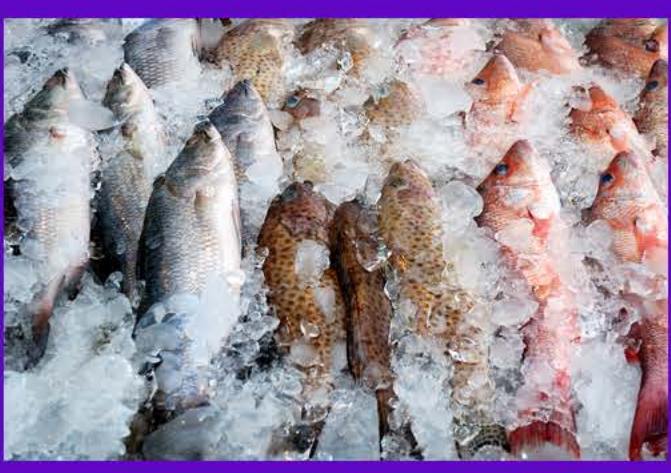

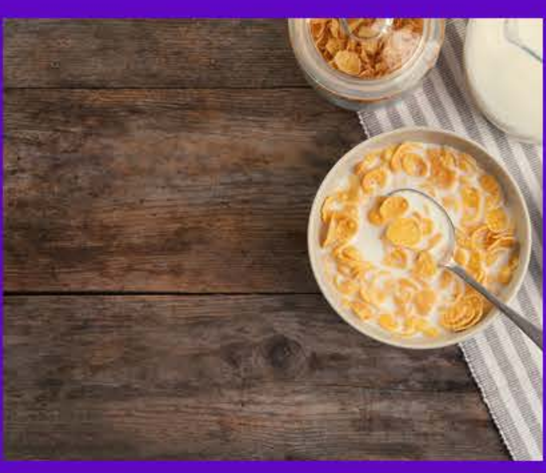

DRAG SLIDER TO REVEAL MORE IMAGES. CLICK IMAGE TO REVEAL MORE INFORMATION

Ready to get started?

Find out if you may be eligible.

SCREENING TOOL →

SNAP Resources | FAQs | Other Food Resources

Disclaimer

All estimates and data on this website are for educational purposes only and provide a basic estimate based on publicly available information. State test, benefit, and allowance amounts may be incomplete, incorrect, or outdated due to limitations in finding updated sources. Eligibility factors vary and eligibility criteria will not be included here. The only way to find out your true eligibility and benefit amount is to apply. This website is not affiliated with any government organization or SNAP program.

Brought to you by: Hunter College New York City Food Policy Center

©2022 HUNTER COLLEGE NEW YORK CITY FOOD POLICY CENTER

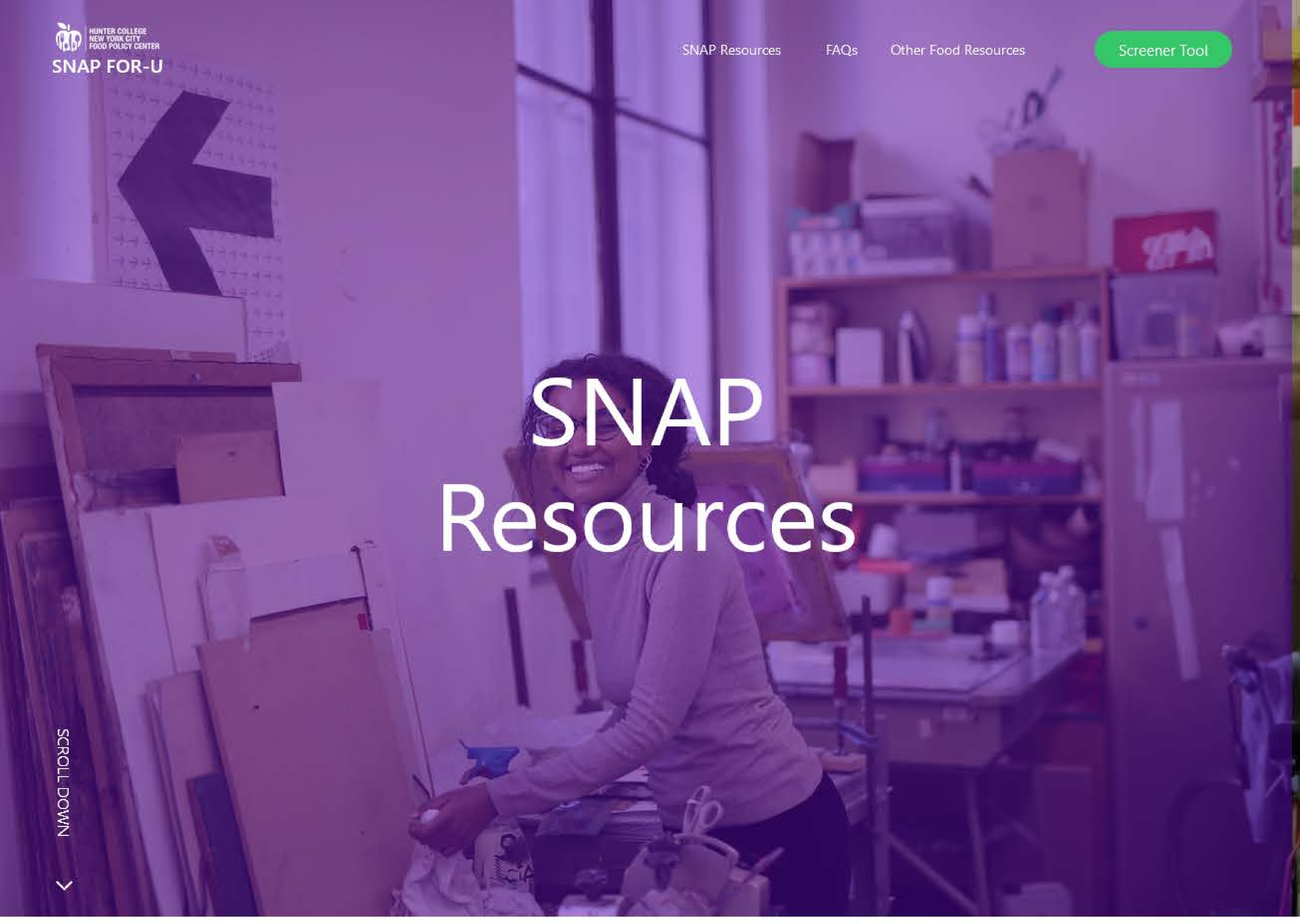

SNAP Resources Guide

Looking to apply for SNAP benefits?

These resources can help guide your next steps.

▾ Step-by-Step Guide to Applying for SNAP and What to Expect.

You will receive SNAP benefits within 30 days of submitting your application if you are determined to be eligible.

1. Create an ACCESS HRA account
2. Fill out the online application on ACCESS HRA
3. Submit documents via the ACCESS HRA app on your phone or mobile device.
   - Documents cannot be submitted from the desktop site, so download the app on your phone or other mobile devices (Android) (IOS) to send in copies of your documents.
   - If you are unable to submit your documents online, you can also go to a SNAP center to submit copies of your documents.
   - Some examples of the documentation that you will need to provide include:
     - Identity/Age Proof: Photo ID, Driver's License, Birth Certificate, U.S. Passport, School ID with date of birth
     - Citizenship status: Birth Certificate, U.S. Passport, Military Service Records, Naturalization Certificate, USCIS Documentation
     - Social Security Number/Proof of SSN Application: Social Security card, Official correspondence from the Social Security Administration
     - Income: Current pay stubs, Pay envelopes, Income tax returns, Current benefit check, Current benefit award letter, Official correspondence from the source of benefits
     - Proof of who lives in your household: Statement from the non-relative landlord, Statement from community organization, Statement from non-household members
     - Residence: Statement from the landlord, Current rent receipt, Current lease, Mortgage records, School records
     - Shelter/Utility Expenses: Current rent receipt, Current lease, Sewer and water bills, Fuel bills, Non-heating utility bills, Telephone bills.
4. Complete an interview.
   - An HRA worker may call you within 5 business days regarding your interview, so be on the lookout for a call from an unfamiliar number.
   - If you do not receive a call or miss the call, you can call (718) SNAPNOW ((718) 762-7669) between 8:30am and 5:30pm, Monday to Friday, for your interview. You may be put on hold for at least 15 minutes to an hour before reaching a representative. Call earlier in the day for quicker wait times.
   - During the interview, you will be asked various questions to confirm the information on your application and on your documents.
5. You can check on the status of your SNAP application from the ACCESS HRA app (Android) (IOS) or on the ACCESS HRA website.

For CUNY students

CUNY SNAP page

This is CUNY's guide to applying for SNAP. You can access information on how to obtain a "Student Verification of Enrollment" on CUNYfirst to provide as documentation with your application.

Learn More

CUNY Food Navigator

This is a free digital referral service for any CUNY student who is looking for help with accessing food and other basic necessities needed to get through these challenging times.

Learn More

For Everyone

Access HRA

With ACCESS HRA, you can apply for some HRA benefits (e.g. SNAP, Cash Assistance, HEAP, Medicaid, and Fair Fares NYC), submit documents for your application, manage your case, and much more!

Learn More

SNAP Application/Recertification Form

This is a printable PDF version of the SNAP Application/Recertification Form.

Learn More

SNAP Centers

Visit a SNAP Center to get assistance with your SNAP application and to access other SNAP services.

Learn More

SNAP Documentation Guide (COVID-19)

This guide provides a list of suggested documentation to provide with your application to determine your SNAP eligibility and benefit amount.

Learn More

Disclaimer

All estimates and data on this website are for educational purposes only and provide a basic estimate based on publicly available information. State test, benefit, and allowance amounts may be incomplete, incorrect, or outdated due to limitations in finding updated sources. Eligibility factors vary and eligibility criteria will not be included here. The only way to find out your true eligibility and benefit amount is to apply. This website is not affiliated with any government organization or SNAP program.

Brought to you by: HUNTER COLLEGE NEW YORK CITY FOOD POLICY CENTER

©2022 HUNTER COLLEGE NEW YORK CITY FOOD POLICY CENTER

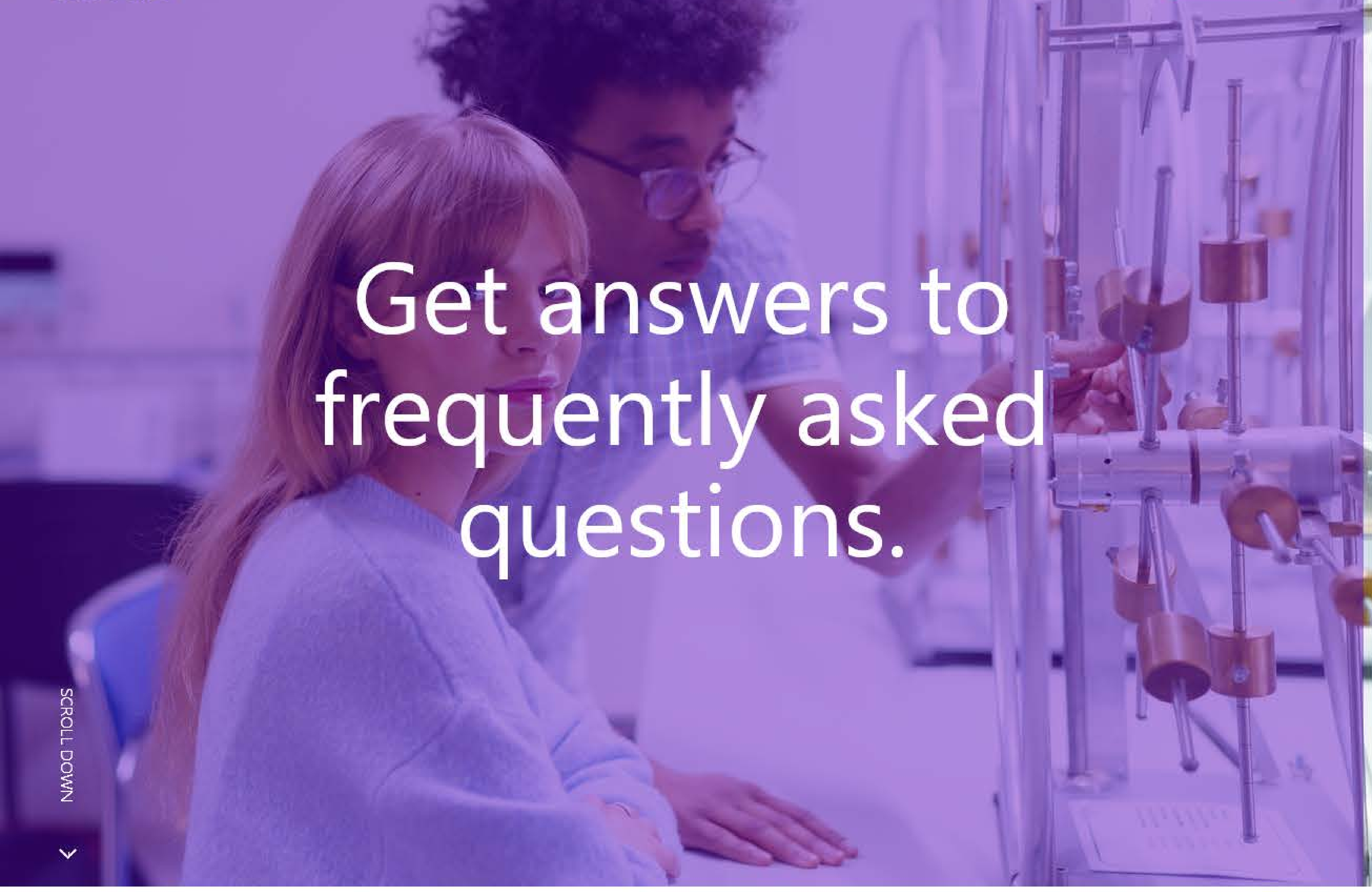

Get answers to frequently asked questions.

SCROLL DOWN

Support

FAQs

Everything you need to know about SNAP assistance. Can't find your answer here? Check out more FAQs **here**

What is SNAP?

The Supplemental Nutrition Assistance Program (SNAP), formerly called Food Stamps, is the largest federal program aimed at combating hunger and food insecurity. SNAP benefits are provided through an Electronic Benefit Transfer (EBT) card, similar to a bank debit card or credit card.

Do I need to meet the required number of work hours?

No. There are other exemption criteria that college students can qualify under instead of working an average of 20 hours per week.

Do I need to have a Social Security Number (SSN) to be eligible for benefits?

What is a "SNAP household"?

I am financially dependent on my parents. Can I still apply for SNAP benefits?

Does financial aid count as income?

Will receiving SNAP benefits affect my immigration status?

Can I check my application's status online?

Do I need to apply in person?

Do I have to be a citizen to be eligible?

I am not the head of my household, but can I apply for SNAP and attend the interview on their behalf?

My parents don't speak English well. Is there any way to get interpreter services to help them apply for SNAP?

I am attending school in a state different from the one of my official residence. In which state should I apply for SNAP benefits?

Disclaimer

All estimates and data on this website are for educational purposes only and provide a basic estimate based on publicly available information. State test, benefit, and allowance amounts may be incomplete, incorrect, or outdated due to limitations in finding updated sources. Eligibility factors vary and eligibility criteria will not be included here. The only way to find out your true eligibility and benefit amount is to apply. This website is not affiliated with any government organization or SNAP program.

Brought to you by: HUNTER COLLEGE NEW YORK CITY FOOD POLICY CENTER

©2022 HUNTER COLLEGE NEW YORK CITY FOOD POLICY CENTER

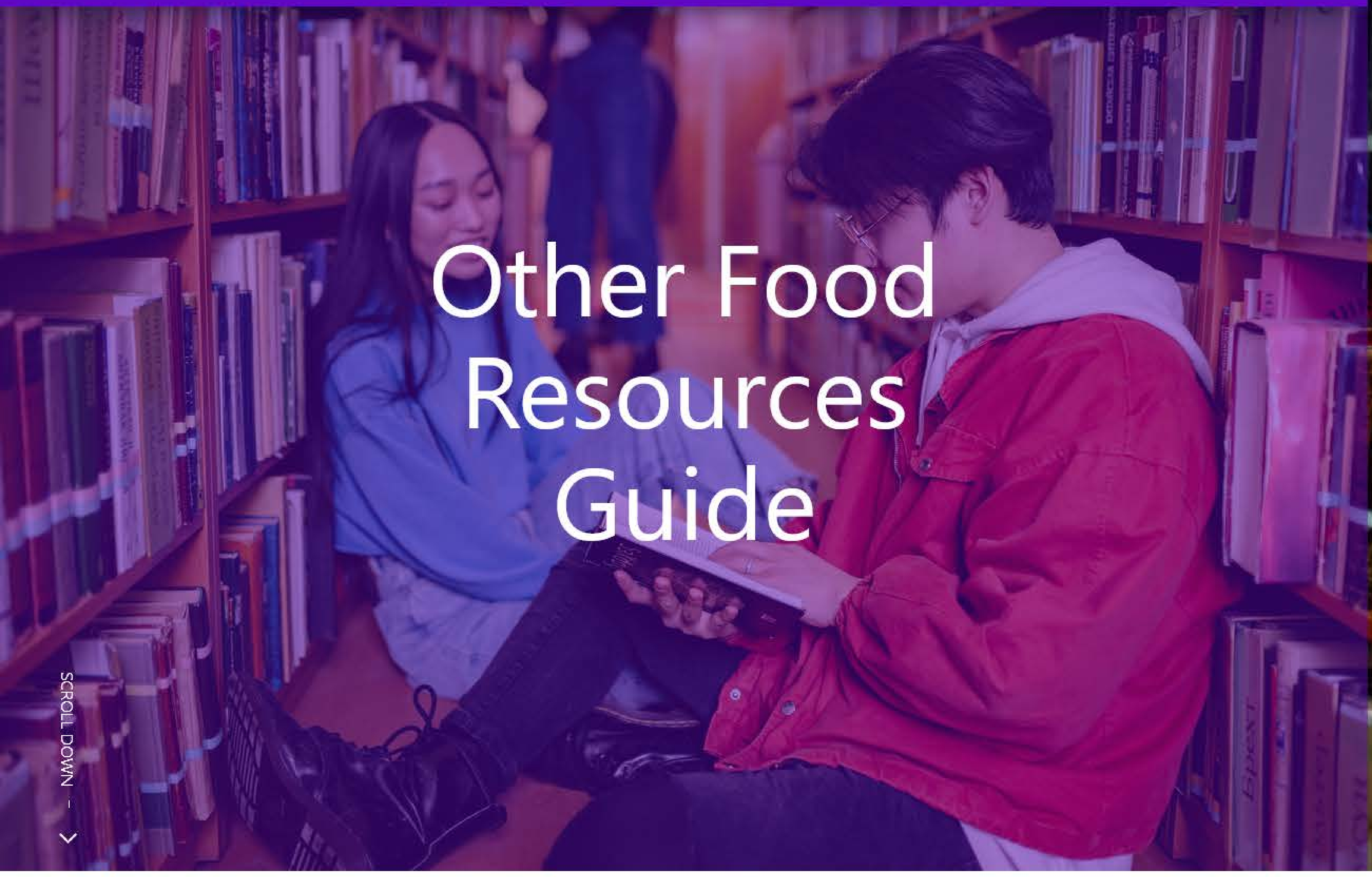

Other Food Resources Guide

SCROLL DOWN –

Food Resources Guide

Check out the collection of food resources below.

Our top recommendations for food resources within the community.

NYC Neighbourhood Food Resource Guides

To help connect community members in need with food resources, the Hunter College NYC Food Policy Center created NYC Neighborhood Food Resource Guides for each NYC neighborhood.

Learn More

Share Meals

Share Meals is dedicated to ensuring all college students are food secure. We empower college students to strengthen their own communities through sharing food and we accomplish this through technology, activism, and advocacy.

Learn More

Food Help NYC

Food Help NYC is an application that can be used to find locations that offer free food (food pantries and soup kitchens). It discloses the hours opened and address of each location, however, these may be subject to change.

Learn More

Too Good To Go

Our mission? To make sure good food gets eaten, not wasted. Every day, delicious, fresh food goes to waste at cafés, restaurants, hotels, shops and manufacturers - just because it hasn't sold in time.

Learn More

Healthy CUNY - Food Pantries

This is a list of all food pantries at CUNY campuses with links to each pantry's webpage.

Learn More

Disclaimer

All estimates and data on this website are for educational purposes only and provide a basic estimate based on publicly available information. State test, benefit, and allowance amounts may be incomplete, incorrect, or outdated due to limitations in finding updated sources. Eligibility factors vary and eligibility criteria will not be included here. The only way to find out your true eligibility and benefit amount is to apply. This website is not affiliated with any government organization or SNAP program.

Brought to you by:
HUNTER COLLEGE NEW YORK CITY FOOD POLICY CENTER

©2022 HUNTER COLLEGE NEW YORK CITY FOOD POLICY CENTER

SNAP
FOR-U

Welcome to SNAP FOR-U!

This screener tool is designed to help you determine your potential eligibility for SNAP benefits and estimate the amount you could receive. Please note this is an unofficial tool and is not affiliated with any government organization. This screener only provides a general estimation based on public information as of August 2022.

At the beginning of the screening questions you can choose to save your progress so that you may stop at any time and continue at a later time. After completing the screening, you will be given the option to email the results to yourself to help you fill out your SNAP application.

This website automatically collects information about how visitors use the website. (e.g. page views, error messages).

We would also like to collect some data to improve this website. Collected information will include submitted answers to the screening tool questions. None of the questions ask for any personal identifiers. The data will not be used to identify any individual and will only be used to improve the website

- [ ] I consent to the collection of my data
- [ ] I do not consent to the collection of my data

GET STARTED →

SNAP
FOR-U

Age

1. * How old are you?

Answer in years.

2. Do you have any disabilities?

This includes physical or mental impairments that severely limits a person's life activities such as: breathing, seeing, hearing, walking, standing, sleeping, caring for yourself, or learning.

Yes No

Clear

Page 1 of 4

Next

Student Status

1. * Are you enrolled in a higher education institution or intend to enroll next semester?

Includes but not limited to: colleges, universities, online courses, vocational/trade/technical schools, any education institution at the post-high school level

- (•) Yes
- ( ) No

2. * What is your enrollment status?

This is defined by your academic institution. Typically, "at least half-time" is considered to be 6 credits or more.

- (•) At least half-time
- ( ) Less than half-time

3. * Are you enrolled in a college meal plan?

- ( ) Yes
- (•) No

Student Exemptions

1. * Which of the following criteria best fits you?

Select ALL that apply.Choices denoted by '*' are temporary exemptions that will remain in effect until COVID-19 is no longer declared a public health emergency.

- [ ] Work an average of 20 hours per week.
- [ ] Have an expected family contribution (EFC) of $0 in the current academic year under federal student financial aid rules.*
- [ ] Eligible to participate in state or federal work-study.*
- [ ] Participate in state or federal work-study.
- [ ] Attend a State University of New York (SUNY) or City University of New York (CUNY) community, comprehensive, or technology college AND is enrolled in a qualified certificate or degree Career and Technical Education (CTE) program.
- [ ] None of the above

Check if your program of study qualifies as a CTE program

Use this to check if your program of study is a Career and Technical Education (CTE) program.

CUNY College Name

Type in your college's name

Remove

Add a program

Page 3 of 6

Previous

Next

Household

1. * How many people are in your household?

Household refers to a group of individuals who live together, AND typically purchase food and prepare meals together for home consumption.

Choose...

Previous

Next

Income

1. * Does your household have earned income?

Earned income refers to income from jobs or self-employment.

(●) Yes ( ) No

2. * What is your household's monthly earned income before taxes?

Earned income refers to income from jobs or self-employment. If you are paid biweekly, multiply your pay by 2. If you receive your paid weekly, multiply it by 4. Enter only the number. Do not include "$" or commas.

Earned Income Calculator

Use this to help you calculate your household's earned income. Remember to enter in the total monthly amount into question 2.

Amount	Frequency	Monthly amount	
	Choose...		Remove
		$0.00	

Add income source

3. What is your household's monthly income from other sources?

Other sources include cash assistance programs, SSI, Social Security, veteran's benefits, unemployment, disability, child support, alimony, bank interest, and cash gifts. Enter only the number. Do not include "$" or commas.

4. Does your household pay for child support?

( ) Yes ( ) No

Clear

5. Monthly Gross Income

This is your calculated total monthly gross income.

0

Deductions/Expenses

1. * Does your household have any of the following expenses?

Select all that apply.

- [ ] Rent or Mortgage
- [ ] Heating or cooling
- [ ] Utilities (e.g. electricity, gas, water, sewer)
- [ ] Phone service (e.g. telephone or cell phone)
- [ ] None

SNAP
FOR-U

Age

1. * How old are you?

23

2. Do you have any disabilities?

This includes physical or mental impairments that severely limits a person's life activities such as: breathing, seeing, hearing, walking, standing, sleeping, caring for yourself, or learning.

- ( ) Yes
- (•) No

Edit

Student Status

3. * Are you enrolled in a higher education institution or intend to enroll next semester?

Includes but not limited to: colleges, universities, online courses, vocational/trade/technical schools, any education institution at the post-high school level

- (•) Yes
- ( ) No

4. * What is your enrollment status?

This is defined by your academic institution. Typically, "at least half-time" is considered to be 6 credits or more.

- (•) At least half-time
- ( ) Less than half-time

5. * Are you enrolled in a college meal plan?

- ( ) Yes
- (•) No

Edit

Student Exemptions

6. * Which of the following criteria best fits you?

Select ALL that apply.Choices denoted by '*' are temporary exemptions that will remain in effect until COVID-19 is no longer declared a public health emergency.

- [ ] Work an average of 20 hours per week.
- [x] Have an expected family contribution (EFC) of $0 in the current academic year under federal student financial aid rules.*
- [x] Eligible to participate in state or federal work-study.*
- [ ] Participate in state or federal work-study.
- [ ] Attend a State University of New York (SUNY) or City University of New York (CUNY) community, comprehensive, or technology college AND is enrolled in a qualified certificate or degree Career and Technical Education (CTE) program.
- [ ] None of the above

Thank you for completing the survey!

You may be eligible for SNAP benefits.

Based on the information you have provided, you may be eligible to receive **$281.00** in SNAP benefits monthly. However, this estimate is for educational purposes only. There are other factors considered to determine eligibility and benefit amounts on the actual SNAP application.

▾ Key Eligibility Factors

- Identity/Age: **Photo ID, Passport, Naturalization Certificate, Birth certificate**
- Residence: **Current lease or rent receipt, Mortgage records**
- Household Size: **Statement from non-relative landlord, Statement from non-household member**
- Income: **Current pay stubs, Income tax returns, Current benefit checks**
- Shelter/Utility Expenses: **Utility bills, Water bills, Telephone bill**
- Citizenship/Immigration: **See more info in FAQs**

Interested in saving a copy of your answers?

Click the button below to submit a copy of your results to your email address.

Enter your email address.

Send Results

Want to apply for SNAP?

Visit NYC's official SNAP information page.

See our SNAP resources for more information.

Interested in other resources?

Check out our food resource guides.

Retake Screener
